# Counteracting roles of MHCI and CD8^+^ T cells in the peripheral and central nervous system of ALS SOD1^G93A^ mice

**DOI:** 10.1186/s13024-018-0271-7

**Published:** 2018-08-09

**Authors:** Giovanni Nardo, Maria Chiara Trolese, Mattia Verderio, Alessandro Mariani, Massimiliano de Paola, Nilo Riva, Giorgia Dina, Nicolò Panini, Eugenio Erba, Angelo Quattrini, Caterina Bendotti

**Affiliations:** 1 Laboratory of Molecular Neurobiology, Department of Neuroscience, IRCCS - Istituto di Ricerche Farmacologiche Mario Negri, Via La Masa 19, 20156 Milan, Italy; 2 Laboratory of Analytical Biochemistry, Department of Environmental Health Sciences, IRCCS - Istituto di Ricerche Farmacologiche Mario Negri, Via La Masa 19, 20156 Milan, Italy; 3 Neuropathology Unit, Department of Neurology, INSPE- San Raffaele Scientific Institute, Dibit II, Via Olgettina 48, 20132 Milan, Italy; 4 Laboratory of Cancer Pharmacology Department of Oncology, Flow Cytometry Unit, IRCCS – Istituto di Ricerche Farmacologiche Mario Negri, via La Masa 19, 20156 Milan, Italy

**Keywords:** Amyotrophic lateral sclerosis, SOD1G93A mice, Neuroinflammation, MHCI, CD8+ T cells, Motor neuron, Peripheral nervous system

## Abstract

**Background:**

The major histocompatibility complex I (MHCI) is a key molecule for the interaction of mononucleated cells with CD8^+^T lymphocytes. We previously showed that MHCI is upregulated in the spinal cord microglia and motor axons of transgenic SOD1^G93A^ mice.

**Methods:**

To assess the role of MHCI in the disease, we examined transgenic SOD1^G93A^ mice crossbred with β2 microglobulin-deficient mice, which express little if any MHCI on the cell surface and are defective for CD8^+^ T cells.

**Results:**

The lack of MHCI and CD8^+^ T cells in the sciatic nerve affects the motor axon stability, anticipating the muscle atrophy and the disease onset. In contrast, MHCI depletion in resident microglia and the lack of CD8^+^ T cell infiltration in the spinal cord protect the cervical motor neurons delaying the paralysis of forelimbs and prolonging the survival of SOD1^G93A^ mice.

**Conclusions:**

We provided straightforward evidence for a dual role of MHCI in the peripheral nervous system (PNS) compared to the CNS, pointing out regional and temporal differences in the clinical responses of ALS mice. These findings offer a possible explanation for the failure of systemic immunomodulatory treatments and suggest new potential strategies to prevent the progression of ALS.

**Electronic supplementary material:**

The online version of this article (10.1186/s13024-018-0271-7) contains supplementary material, which is available to authorized users.

## Background

Amyotrophic lateral sclerosis (ALS) is the most common neuromuscular disorder, affecting individuals from all ethnic backgrounds, with an incidence of 2–3 cases per 100,000 individuals per year [1, 2]. The pathology, causing a progressive motor neuron (MN) loss and muscle denervation, results in progressive paralysis and death, usually due to respiratory failure [1, 2]. The average patient’s lifespan ranges between 2 and 5 years after diagnosis [2].

Genetic factors contribute to the disease in 10% of all ALS cases corresponding to the familial form [3]. Although more than 30 genes have been associated with familial ALS yet, transgenic mice overexpressing mutant human Cu/Zn dependent SOD1 (mSOD1) are currently the animal model that best mimics some phenotypical and pathological features of both familial and sporadic ALS [2, 4]. There is growing evidence of a prominent role of the immune system in the pathogenesis and progression of ALS [5–9].

Adaptive and innate immune cell infiltrate the CNS of ALS patients [10] and in the CNS [6–8] and peripheral nervous system (PNS) of mSOD1 mice [11, 12] at different stages of the disease. The role of immunity is multifaceted with different cell types influencing the disease progression and the same cell type having a positive or negative effect depending on the disease stage [9]. This may explain why immunosuppressive treatments used in different clinical trials were not effective and in some cases even detrimental [13, 14]. For example, CD4^+^ T cells, in particular, CD4^+^-FoxP3 T cells, are recruited to the sites of damage in mSOD1 mice to protect MNs by maintaining an anti-inflammatory milieu during the early stable phase of the disease [7, 15]. In contrast, CD8^+^ T-cells (cytotoxic T lymphocytes; CTLs) infiltrating the CNS of ALS patients and mSOD1 mice [8, 10, 16, 17] have been classically considered detrimental for MNs. This is because CTLs are antigen-specific effector cells that express the ligand for Fas (FasL) [18] and MNs expressing ALS-linked SOD1 mutations showed enhanced susceptibility to Fas-mediated death in vitro [19, 20]. Moreover, mSOD1 mice with homozygous loss-of-function FasL mutation present a reduced MN loss and prolonger life expectancy [21]. These data indicate that CTLs may contribute to exacerbating the neuromuscular damage, but this hypothesis has never been adequately verified in mSOD1 mice.

We previously found that the immunoproteasome and the major histocompatibility complex I (MHCI), responsible for the generation and presentation of antigen peptides to CTLs, respectively, were highly expressed in the spinal cord and peripheral motor axons of mSOD1 mice [11, 22–25]. Surprisingly, MHCI signaling and CTLs infiltrates were higher in the periphery of mice with slower denervation and progression of disease than mouse with fast disease progression [11, 22]. We therefore hypothesized that the extent of expression of MHCI and CTLs infiltration in the PNS might influence the variability in disease progression in mSOD1 mice, suggesting a potential protective role of the MHCI-related process [5, 11, 26].

Song et al. [27] also showed that the sustained expression of MHCI in MNs protects them from ALS astrocyte–induced toxicity and delays disease progression in mSOD1 mice. However, it was not been addressed whether the protective action of MHCI in vivo was independent of the interaction with the CTLs. Therefore, we investigated if the depletion of MHCI-dependent CTLs activity in mSOD1 mice had a detrimental or beneficial effect on MN viability and disease progression. For this purpose, we produced C57SOD1^G93A^ mice defective for MHCI cell-surface expression and CTLs.

This lack resulted in acceleration of the motor onset due to the increase of hindlimb muscle denervation. However, the MN somata were protected in the spinal cord especially at the cervical level resulting in significant delay in the forelimbs impairment which led to an extension of survival. This suggested that the activation of MHCI in the PNS of ALS mice is an early protective response directed to the preservation of muscle innervation and motor function. Whereas, in the CNS the interaction of microglia expressing MHCI with CD8^+^ T cells accelerates MN death and reduces the overall survival of SOD1^G93A^ mice.

## Methods

### Animals

C57BL6.129P2-B2mtm1Unc/J (stock no: 002087; Jackson Laboratories) females were crossed with C57BL/6JSOD1^G93A^ (stock no: 002726; Jackson Laboratories) male mice, expressing approximately 20 copies of human mutant SOD1 with a Gly93Ala substitution to obtain transgenic mice null for the β2m subunit. Female mSOD1 mice with or without β2m and the corresponding NTG littermates were used for the analysis. Procedures involving animals and their care were conducted according to the Mario Negri institutional guidelines. The Institute adheres to the principles set out in the following laws, regulations, and policies governing the care and use of laboratory animals: Italian Governing Law (D.lgs 26/2014; Authorisation n.19/2008-A issued March 6, 2008 by Ministry of Health); Mario Negri Institutional regulations and Policies providing internal authorisation for personsconducting animal experiments (Quality Management System Certificate- UNI EN ISO 9001:2008 - Reg. N° 6121); the NIH Guide for the Care and Use of Laboratory Animals (2011 edition) and EU directives and guidelines (EEC Council Directive 2010/63/UE). The Statement of Compliance (Assurnace) with the Public Health Service (PHS) Policy on Human Care and Use of Laboratory Animals has been recently reviewed (9/9/2014) and will expire on September 30, 2019 (Animal Welfare Assurnace #A5023–01). Mice were maintained at a temperature of 22 ± 2 °C with a relative humidity 55 ± 10% and 12 h of light / dark cycle. Food (standard pellets) and water were supplied ad libitum.

### Disease progression and survival

Disease progression was monitored bi-weekly, starting from ten weeks of age, in SOD1^G93A^ transgenic mice wild-type and knockout for β2 microglobulin, and their respective NTG littermates,. Body weight and paw grip strength were recorded for each session, as previously described [28]. The Paw Grip Endurance (PaGE) test involved placing the mouse on the wire-lid of a conventional housing cage. For this analysis, the mice are placed on a horizontal grid at about 30 cm from the table and the tail is gently pulled until they grasp the grid with their fore and hind paws. The lid is then gently turned upside down and the latency time of the mouse to fall on the table is recorded for a maximum of 90 s. Each mouse is given up to three attempts and the longest latency is recorded. The onset of hindlimb force deficit is considered when the mice showed the first signs of impairment (latency less than 90 s) in PaGE test. The disability onset is when the mouse for the first time is unable to perform the PaGE test. The mice are euthanized when they are unable to right themselves within ten seconds after being placed on each side according to the institutional ethical committee guidelines. The age at the euthanasia was considered as time of survival. Disease duration was calculated as the difference in days between the onset of hindlimb impairment and the age of death. Days of survival after the onset of disability is the difference in days between the age when the animal is entirely unable to perform the PaGE test and the age at euthanasia. All tests were done by the same operator blinded to the mouse genotype.

### Immunohistochemistry

Spinal cord and sciatic nerve and muscles were processed as previously described [22]. Briefly, mice were perfused with Tyrodes’s buffer, followed by Lana’s fixative (4% formalin and 0.4% picric acid in 0.16 M PBS, pH 7.2) at 20 °C, and tissues were quickly dissected out. The tissue was left in the same fixative for 180 min at 4 °C, rinsed, and stored 24 h in 10% sucrose with 0.1% sodium azide in 0.01 M PSB at 4 °C for cryoprotection, before mounting in optimal cutting temperature compound (OCT).

Unless otherwise specified, the following primary antibodies and staining were used: rat anti-MHC class I ER-HR 52 clone (1:100; Abcam); mouse anti-GFAP (1:2500; Millipore); rabbit anti-NF200 (1:200; Sigma-Aldrich); mouse anti-vimentin (1:250; Millipore); mouse anti-phosphorylated neurofilament H (Smi31; 1:5000; Sigma); Neurotrace conjugated with Alexa-647 (1:500; Invitrogen); goat anti p75^NTR^ (1:200; Santa Cruz Biotech). Alexa- 488, 594 and 647 secondary antibodies (Invitrogen) were used with a dilution of 1:500. All immunohistochemistry was done following an indirect immunostaining protocol.

Spinal cord immunohistochemistry was done on free-floating sections (30 μm), then mounted on glass slides (Waldemar Knittle) with 1:1 PBS 0.1 M: glycerol. Longitudinal sections of sciatic and radial nerves (14 μm) were treated directly on poly-lysine objective slides (VWR International) as described below, then mounted with 1:1 PBS 0.1 M: glycerol. Fluorescence-labeled samples were analyzed under a sequential scanning mode to avoid bleed-through effects with an IX81 microscope equipped with a confocal scan unit FV500 with three laser lines: Ar-Kr (488 nm), He-Ne red (646 nm), and He-Ne green (532 nm) (Olympus, Tokyo, Japan) and a UV diode using a 10X objective (zoom 1,5×).

### Motor neuron impairment

The number of MNs was determined on serial sections (one every ten sections) from lumbar spinal cord segments L2-L5 and cervical spinal cord segments C1-C8 for each mouse. The sections were stained with cresyl violet to detect the Nissl substance of neuronal cells. A total of 12 serial sections were acquired with a CCD color camera (Color View III; Soft Imaging System GmbH) at 10X, using AnalYSIS software (Soft Imaging Systems GmbH, ver. 3.2) and neuron areas were analyzed with Fiji software (Image J, U. S. National Institutes of Health, Bethesda, Maryland, USA). Only neuronal somas with an area ≥ 400 μm^2^ were considered for quantitative analysis of MN numbers.

MN impairment was evaluated on serial sections from lumbar and cervical spinal cord segments for each mouse. Six sections per animal were acquired under the laser scanning confocal microscope (Olympus, Tokyo, Japan) using a 20X objective, and analyzed using Fiji software (Image J, U. S. National Institutes of Health, Bethesda, Maryland, USA) to determine the percentage of MNs with an area ≥ 400 μm^2^ (identified by Neurotrace) immunostained with Smi31.

### Immunohistochemical analysis of MHCI, GFAP, and p75^NTR^ in sciatic and radial nerves

After 0.1 M PBS perfusion, radial and sciatic nerves were dissected out from the same animal and mounted in OCT. Serial longitudinal sections (14 μm) were collected on poly-lysine objective slides (VWR International). For each slice, fluorescence fields were taken the laser scanning confocal microscope (Olympus, Tokyo, Japan). The mean grey value of the immunoreactivity was assessed through Fiji (Image J, U. S. National Institutes of Health, Bethesda, Maryland, USA) for each section in the analysis.

### Muscle denervation and endplates

*Tibialis anterior* and *triceps brachii* were dissected out, and snap-frozen in isopentane cooled in liquid nitrogen. 20-μm serial longitudinal cryosections were collected on poly-lysine objective slides (VWR International). Five serial sections (average ~ 70 NMJs) per animal were analyzed. Muscle sections were stained with anti-synaptic vesicle protein (SV2; 1:100; Developmental Studies Hybridoma Bank), mouse anti-neurofilament 165 kDa (2H3; 1:50; Developmental Studies Hybridoma Bank), followed by 647 anti-mouse secondary antibody (1:500; Invitrogen). α-Bungarotoxin coupled to Alexa Fluor 488 (1:500) (Invitrogen) was then added and left for 2 h at room temperature.

Innervation analysis was performed directly. Images of all genotypes for the innervation analysis were obtained with an Olympus virtual slide system VS110 (Olympus, Center Valley, PA, USA) at 40X-magnification. Images for endplate size analyses were captured with an epifluorescence microscope system (Axio Imager M1 Upright microscope, Zeiss) at 40× magnification with Q-capture software. The percentage of neuromuscular innervation was quantified in OlyVIA (Olympus) on the basis of the overlay between neurofilament (SV2/2H3) staining and α-BTX labeled endplates. Endplates were quantified as occupied when there was any neurofilament staining overlying the endplate and as vacant when there was no overlay. Endplate area was determined using Fiji software (ImageJ, National Institutes of Health). Endplates were manually outlined, and the area was measured. Diaphragm: after excision, tissues were stretch over silicone rubber to make it taut, using insect pins, in a glass 100 mm Petri dish, fixed in 4% paraformaldehyde for 4 h and stored 24 h in 30% sucrose with 0.1% sodium azide in 0.01 M PSB at 4 °C for cryoprotection. After this, connective tissue was cleaned off using a stereomicroscope and the right and left muscle areas were cut into pieces before mounting in OCT; 20-μm serial longitudinal cryosections were collected on poly-lysine objective slides (VWR International). At least five serial sections per animal were analyzed. Muscle sections were stained with anti-Synaptophysin (1:100; Synaptic system), followed by 488 anti-mouse secondary antibody (1:500; Invitrogen). α-Bungarotoxin coupled to Alexa Fluor 594 (1:1000) (Invitrogen) was then added and left for 15′ at room temperature. For each slice, consecutive fluorescence fields along the z-axis were taken using the laser scanning confocal microscope (Olympus, Tokyo, Japan) using a 20X objective (zoom 2×) at 0.43 μm intervals Denervation was analysed using Imaris 7.4.2 (Bitplane). The colocalization channel between Synaptophysin and BTX immunostaining was produced for each Z-stack. Then, rendering in iso-surfaces was done on the colocalization and BTX channels, and the ratio in voxels (μm^3^) was calculated.

### Morphometric analysis of muscles and sciatic nerves

Tibialis anterior muscles were dissected out and snap-frozen in isopentane cooled in liquid nitrogen. Muscle fiber architecture and composition were analyzed by hematoxylin and eosin (H&E) and nicotinamide adenine dinucleotide tetrazolium reductase (NADH-TR) staining. Serial transverse cryosections (12 μm) from the mid-belly region of the tibialis anterior muscle were mounted on poly-lysine objective slides (VWR International). For H&E staining, sections were air-dried and fixed in 4% paraformaldehyde solution for 5‘, washed in water and stained with hematoxylin (Merck) for 5’. After bluing, sections were stained with 0.5% eosin solution (Merck) containing 1% acetic acid for 10′ and washed. After dehydration in a graded series of alcohol (70, 90, 100%) and clearing in 100% xylene, sections were mounted with DPX compound (Sigma Aldrich). For NADH staining, sections were air-dried then incubated at 37 °C for 30′ in Tris-HCl buffer (50 mM, pH 7.4) containing 0.4 mg/mL β-NAD reduced disodium salt hydrate (Sigma-Aldrich, St. Louis, MO, USA, 0.71 mg/mL buffer solution) and 1 mg/mL nitro blue tetrazolium (Sigma-Aldrich, 0.29 mg/mL buffer solution). After staining, sections were fixed with 4% paraformaldehyde, dehydrated in a graded series of alcohol (70, 90, 100%), cleared in 100% xylene and finally mounted with DPX compound (Sigma Aldrich). For both applications, images were acquired with a CCD color camera (Color View III; Soft Imaging System, GmbH), using AnaliSYS software (Soft Imaging Systems, GmbH, ver. 3.2) at 10X and 20X- magnification for H&E and NADH staining, respectively.

Muscle fiber CSA, number, and density were analyzed with Fiji (Image J, U. S. National Institutes of Health, Bethesda, Maryland, USA) as previously described [29]. Briefly, a grid of rectangular sampling fields was outlined on the muscle slice profile. To ensure that every part of the slice had an equal chance of being sampled, a systematic random sampling procedure was applied considering rectangular field placed at a fixed distance from each other using the “Grid” function in Fiji. Respectively, four and two serial cryosections for each mouse were analyzed for H&E and NADH. For the morphometric analysis of axons, sciatic nerve samples were fixed with 4% PFA and 2% glutaraldehyde in 0.12 M PBS and post-fixed with 1% OsO4 in 0.12 M cacodylate buffer, dehydrated in graded series of ethanol, and embedded in epoxy resin (Fluka). Coronal semithin sections (1 um), were stained with 0.1% toluidine blue in 0.12 M phosphate buffer. The images were acquired with an Olympus virtual slide system VS110 (Olympus, Center Valley, PA, USA) at 20X-magnification. Diameter and caliber of axons were assessed through Fiji (Image J, U. S. National Institutes of Health, Bethesda, Maryland, USA) on three serial sections per animal with the same procedure described above.

### Flow cytometric analysis

At 70, 123 and 140 d 25 μL of whole blood were collected in EDTA 10 mM and Polybrene 0.125% from the submandibular plexus of anesthetized mice. Samples were incubated with 600 μL ACK lysing buffer (Lonza) to lyse red blood cells. After centrifugation (1,4 rcf at 4 °C for 7 min), the ACK solution was removed, and the pellet was washed twice with cold PBS + 1% FBS (FACS buffer). The pellet was then incubated for 30 min at 4 °C in the dark in 100 μL of FACS buffer with the following primary monoclonal antibodies: FITC-labeled rat anti-mouse CD3ε (BD Pharmingen), Cy5.5-labeled rat anti-mouse CD8 α-chain (BD Pharmingen); APC-labeled rat anti mouse CD4 α-chain (BD Pharmingen). Each flow cytometric analysis was run on at least 10,000 cells on a Gallios flow cytometer (Beckman Coulter) equipped with 488, and 638 nm lasers and the data were analyzed using Kaluza software.

### Western blot

After deep anesthesia, mice were decapitated and sciatic nerve and muscles were rapidly dissected, frozen on dry ice and stored at − 80 °C. The samples were powdered in liquid nitrogen then homogenized by sonication in ice-cold homogenization buffer (Tris HCl pH 8 50 mM, NaCl 150 mM, EGTA pH 8.5 mM, MgCl2 1.5 mM, Triton x-100 1%, anhydrous glycerol 10%, NaF 50 mM, NaPP 10 mM, Na_3_VO_4_ 10 mM, PMSF 0,1 mg/mL, leupeptin 0,02 mg/mL, aprotinin 0.02 mg/mL, DTT 1 mM), centrifuged at 13000 rpm for 15 min at 4 °C and the supernatants were collected and stored at − 80 °C.

Equal amounts of total protein homogenates were loaded on polyacrylamide gels and electroblotted onto PVDF membrane (Millipore) as previously described [22]. Membranes were immunoblotted with the following primary antibodies: mouse anti β-actin (1:30000; Chemicon); mouse anti Importin β; (1:5000; Millipore); mouse anti β^III^-tubulin (1:1000; Millipore); rabbit anti ERK (1:1000; Santa Cruz Biotech); mouse anti phospho-ERK (1:1000; Santa Cruz Biotech); rabbit anti NF200 (1:1000; Sigma-Aldrich); mouse anti GFAP (1:10000; Millipore); goat anti p75^NTR^ (1:1000; Santa Cruz Biotech); rabbit anti S100β (1:200; Sigma Aldrich); rabbit anti AChR-α7 (1:100; Millipore); rabbit anti NCAM (1:2000; Millipore); mouse anti GAPDH (1:10.000; Millipore); mouse anti MBP (1:1000; R&D); followed by HRP-conjugated secondary antibodies (Santa Cruz) and developed with Luminata Forte Western Chemiluminescent HRP Substrate (Millipore) on the Chemi-Doc XRS system (Bio-Rad). Densitometric analysis was done with Progenesis PG240 v2006 software (Nonlinear Dynamics). Immunoreactivity (IR) was normalized to β-actin, GAPDH or to the total amount of protein detected by red Ponceau (Sigma Aldrich) as previously published [22]. When necessary, more than one membrane was analysed as follows: i) an internal standard (IS) representing the mix of all the samples in the experiment was loaded on each gel; ii) membranes were acquired at the same time; iii) the immunoreactivity of each sample was further normalized to the immunoreactivity of the IS.

### Real-time PCR

Tissues (spinal cords, sciatic nerves, and muscles) were freshly collected and immediately frozen on dry ice after mouse perfusion with 0.1 M PBS. The total RNA from spinal cord was extracted using the Trizol method (Invitrogen) and purified with PureLink RNA columns (Life Technologies). For fibrous tissues (sciatic nerve and muscles), the RNeasy® Mini Kit (Qiagen) was used. RNA samples were treated with DNase I and reverse transcription was done with a High Capacity cDNA Reverse Transcription Kit (Life Technologies). For Real-time PCR we used the Taq Man Gene expression assay (Applied Biosystems) following the manufacturer’s instructions, on cDNA specimens in triplicate, using 1X Universal PCR master mix (Life Technologies) and 1X mix containing specific receptor probes. The following probes were used for the real-time PCR: CD8 alpha receptor (*CD8*; Mm01182107_g1; Life Technologies); CD4 alpha receptor (*CD4*; Mm00442754_m1); Forkhead box P3 (*FoxP3*; Mm00475162_m1); cholinergic receptor nicotinic, gamma subunit (CHRNG; Mm00437419_m1; Life Technologies); insulin growth factor 1 (*Igf1*; Mm00439560_m1); Interferon-γ (*Ifnγ*; Mm01168134_m1; Life Technologies) monocytes chemoattract protein-1 (*Ccl2*; Mm00441242_m1; Life Technologies); CD68 (*Cd68*; Mm03047343_m1; Life Technologies); interleukin 1β (*Il-1β*; Mm01268569_m1; Life Technologies); interleukin 23 (*Il-23*; Mm00519943_m1; Life Technologies). Relative quantification was calculated from the ratio between the cycle number (Ct) at which the signal crossed a threshold set within the logarithmic phase of the given gene and that of the reference β-actin gene (4310881E; Life Technologies). Mean values of the triplicate results for each animal were used as individual data for 2^-ΔΔCt^ statistical analysis.

### In vitro analysis of motor neuron loss and microglia activation in primary co-cultures

Primary cultures were obtained from the spinal cord of 13-day-old (E13) NTG+/+ or NTG−/− mouse embryos, as previously described [30]. Briefly, ventral horns were dissected from spinal cords, exposed to DNAse and trypsin (Sigma-Aldrich) and centrifuged with a bovine serum albumin (BSA) cushion. Cells obtained at this step were a mixed neuron/glia population and were centrifuged (800 *g* for 15 min) through a 6% iodixanol (OptiPrep™; Sigma-Aldrich) cushion for motor neuron enrichment. A sharp band (motor neuron-enriched fraction) at the top of the iodixanol cushion and a pellet (glial fraction) were obtained. The glial feeder layer was prepared by plating the glial fraction at a density of 25,000 cells/cm^2^ into flasks already pre-coated with poly-L-lysine (Sigma-Aldrich). Flasks containing confluent mixed glial cultures were shaken overnight at 275 rpm in incubators to obtain purified microglia cultures. The supernatants containing microglial cells from NTG+/+ or NTG−/− mouse embryos were collected and seeded at a density of 40,000 cells/cm^2^ in 24-well plates for mRNA expression analysis or added (10% of the astrocyte number) to astrocyte cultures the day before MN sowing. NTG+/+ astrocyte-enriched cultures were obtained by treating the glial cultures from which microglia had been harvested with 60 mmol/L L-leucine methyl ester (Sigma-Aldrich) for 90 min. To prepare a feeder layer for “sandwich” co-cultures, astrocytes were collected and seeded at a density of 25,000 cells/cm^2^ into 12-well plates.

To establish neuron/glia cocultures, the NTG+/+ motor neuron-enriched fraction (from the iodixanol-based separation) was seeded at a density of 10,000 cells/cm^2^ onto mature glial layers composed of mixed glial cells (NTG+/+ astrocyte plus NTG+/+ or NTG−/− microglia).

Culture treatments: Primary cultures were exposed to 1 μg/mL LPS (from *Escherichia coli* 0111:B4) on the fifth-sixth day in vitro (5–6 DIV) for 24 h. Cultures maintained with normal medium served as the control condition. As reported below, MN viability was assessed by counting SMI32-positive cells in each treatment condition, and microglia activation was analyzed considering different cell morphology parameters and the gene expression of pro-inflammatory cytokines. Immunocytochemical and immunofluorescent Assays: cells were fixed with 4% paraformaldehyde and permeabilized by 0.2% Triton X-100 (Sigma-Aldrich). Staining was carried out by overnight incubation with the primary antibody, followed by incubation with an appropriate fluorescent secondary antibody for immunofluorescence (Dy-light; Rockland Immunochemicals). Double staining was done by overnight incubation of the cultures separately with each primary antibody. In each experiment, some wells were processed without the primary antibody to verify the specificity of the staining. Primary antibodies were: mouse anti-nonphosphorylated neurofilament H (SMI32, 1:1000; Covance). Appropriate fluorescent secondary antibodies conjugated to different fluorochromes were used at 1:1000 dilution. Pictures of stained cells were obtained with an Olympus virtual slide system VS110 (Olympus, Center Valley, PA, USA) at 10X-magnification, and images were analyzed with Fiji (Image J, U.S. National Institutes of Health).

MN viability: The viability of MNs was assayed by counting SMI32-positive cells with typical morphology (triangular shape, single well-defined axon) and intact axons and dendrites, considering five non-overlapping 2 × 12-mm fields (total area analysed: about 30% of each well). This number was normalized to the mean of SMI32-positive cells counted in the appropriate control wells. Microglia activation: to determine the activation status of NTG+/+ or NTG−/− immunocompetent cells after LPS treatment, mixed neuron/glia cocultures were examined by immunocytochemistry with rabbit anti Iba-1 (1:200; Wako), while purified cultures of microglia were analyzed for the gene expression of pro-inflammatory cytokines (IL-23 and IL-1β). Images of Iba-1-positive cells were obtained with an Olympus virtual slide system VS110 (Olympus, Center Valley, PA, USA) at 100X magnification, and the morphological parameters (cell area and circularity) were measured with Fiji (Image J, U.S. National Institutes of Health) considering from four to eight non-overlapping stereological 2 × 12-mm fields. For mRNA analysis, we harvested microglia cell cultures and extracted the mRNA following the approach described in the “Real-time PCR” section.

### Statistical analysis

GraphPad v7.03 (GraphPad Software) was used. The Mantel-Cox log rank test was used for comparing disease onset and survival between groups. Paw Grip Strength and body weight were analyzed by repeated measures ANOVA with Sidak’s post analysis. The unpaired t-test was used to compare differences between two groups. One-way ANOVA with Tukey’s post analysis was used to compare differences between more than two groups. Further details are provided in the captions.

## Results

### The lack of MHCI and CTLs accelerates the symptoms onset but extends the survival in mSOD1 mice

**Fig. 1:**
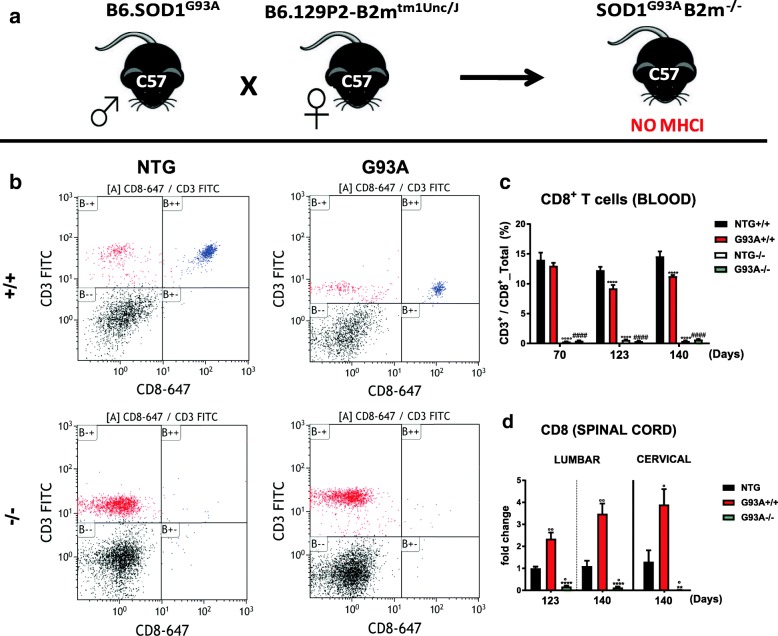
MHCI depletion affects the production and the infiltration of CD8^+^ T cells in mSOD1 mice. **a** Schematic representation C57BL6.129P2-B2mtm1Unc/J females bred with C57BL/6JSOD1^G93A^ males mice in order to obtain transgenic mice null for β2microglobulin. **b** Representative FACS scatter plots of CD3^+^/CD8^+^ T cells in the peripheral blood of NTG+/+ mice, G93A+/+, NTG−/− and G93A−/− mice at 123 d. **c** Longitudinal FACS measurement of the percentage of CD3^+^/CD8^+^ T cells in the peripheral blood of G93A+/+; G93A−/− mice and relative controls at 70, 123 and 140 d. Data are reported as the mean ± SEM of six independent experiments (6 mice) for G93A+/+ and NTG+/+ mice and eight independent experiments (8 mice) for G93A−/− and NTG−/− at each time point. ^****^P < 0.0001 (G93A+/+ vs NTG+/+); ^°°°°^*P <* 0.0001 (NTG−/− vs NTG+/+ and G93A+/+); ^####^*P <* 0.0001 (G93A−/− vs NTG+/+ and G93A+/+). **d** Real-time PCR for the CD8α receptor transcript in the lumbar and cervical spinal cord of G93A+/+, G93A−/− mice compared to NTG +/+ littermates at 123 and 140 d. Data are normalized to β-actin and expressed as the mean ± SEM fold change ratio between G93A+/+ mice, G93A−/− mice and control mice from four independent experiments for each genotype at both stages. ^**^P < 0.05; ^****^P < 0.0001 (G93A+/+ vs G93A−/−); ^°^*P < 0.05;* ^°°^*P < 0.01;* (G93A−/− or G93A+/+ vs NTG) by one-way ANOVA with Tukey’s post-analysis

Mice homozygous for the *β2m*^*tm1Unc*^ targeted mutation (B6.129P2-*β2m*^*tm1Unc*^/J mice) lacking *β2m* produce minimal, if any, MHCI presentation on the cell surface [31]. They have no mature CD8^+^ T cells and do not present CD8^+^ T cell-mediated toxicity [31, 32]. We crossed female mice homozygous lacking *β2m* with C57SOD1^G93A^ transgenic male mice and examined their F1 progeny (Fig. 1a). To accurately assess the level of CD8^+^ T cells in SOD1^G93A^ mice, we did a longitudinal FACS analysis on the peripheral blood of SOD1^G93A^β2M^−/−^ (**G93A−/−**) mice, SOD1^G93A^β2M^+/+^ (**G93A+/+**) mice and relative non-transgenic (NTG) littermates (**NTG+/+**; **NTG−/−**) during the disease progression (70 d = presymptomatic, 123 d = motor onset; 140 d = symptomatic stage). Blood CD3^+^-CD8^+^ lymphocytes in G93A+/+ mice at age 123 and 140, but not 70 d, were significantly lower than in NTG littermates (Fig. 1b, c). In contrast, the expression level of the CD8α receptor in the lumbar spinal cord of the same G93A+/+ mice at 123 and 140 d was significantly higher than in NTG littermates, and the same was found in the cervical spinal cord of G93A+/+ mice at 140 d (Fig. 1d). This agrees with the possible recruitment of these cells in the CNS from the systemic circulation. However, both NTG and mSOD1 mice lacking MHCI/CTLs (**NTG−/−; G93A−/−**) had negligible hematogenous CD3^+^-CD8^+^ lymphocyte counts at all time-points (Fig. 1b, c), with complete depletion of CD8α receptor mRNA in the spinal cord (Fig. 1d).

We next evaluated the blood levels of CD4 T+ cells in both G93A+/+ and G93A−/− mice and relative controls. In keeping with the literature [31], we found that Ntg^−/−^ and G93A^−/−^ mice compensated for the lack of CD8+ T cells by increasing the blood expression of CD3^+^-CD4^+^ lymphocytes with respect to Ntg+/+ and G93A+/+, at all time-points considered (Additional file 1: Figure S1a, b). However, this did not translate in a higher infiltration of CD4^+^ T cells within the spinal cord since no difference was found in the levels of the CD4 receptor, FoxP3 and FoxP3/CD4 ratio between G93A+/+ and G93A^−/−^ mice at both 123 d and 140 d (Additional file 1: Figure S1c-f).

**Fig. 2:**
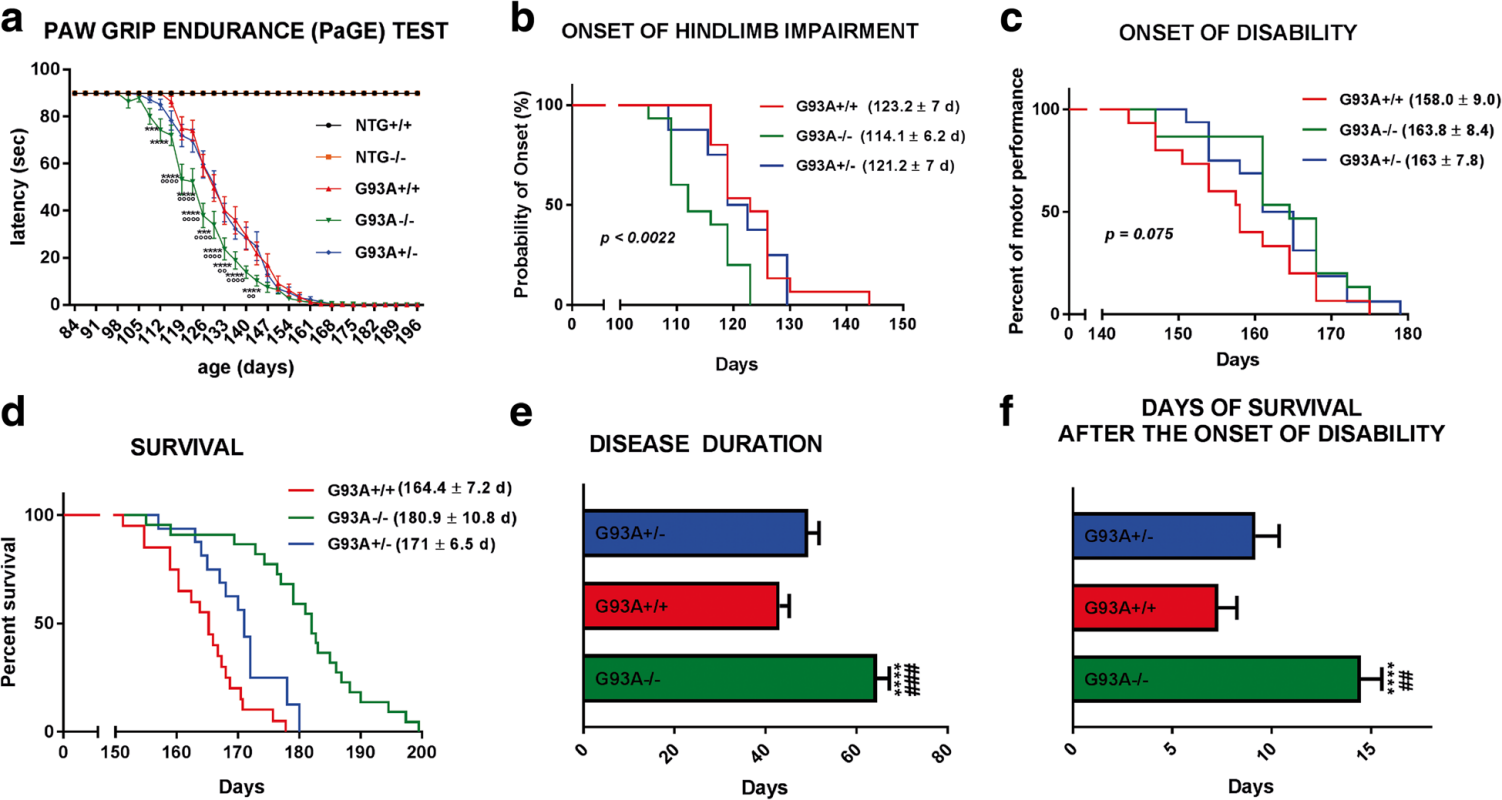
MHCI and CTLs depletion accelerate disease onset and motor deficits but increases survival in mSOD1 mice. **a** Paw Grip Endurance (PaGE) test for Ntg*+/+,* Ntg*−/−*, G93A+/+ and G93A^−/−^ mice. Data are reported as mean ± SEM for each time point. ^***^P < 0.001; ^****^P < 0.0001 (G93A−/− vs G93A+/+); ^°°^*P < 0.001*; ^°°°°^*P* < 0.0001 (G93A−/− vs G93A+/−) by repeated measures ANOVA with Sidak’s post-analysis. **b** G93A−/− mice have an earlier onset of motor impairment than G93A^+/+^ and G93A+/− mice. *P* < 0.0022 by Mantel-Cox log-rank test. **c** G93A^−/−^ mice display a trend to have delayed the onset of disability compared to G93A+/+. (*P* = 0.075 by Mantel-Cox log-rank test. **d** G93A^−/−^ mice display a prolonged survival in respect to G93A^+/+^ and G93A+/− mice. *P* < 0.0001 by Mantel-Cox log-rank test. **e** G93A^−/−^ mice have longer disease duration than G93A+/+ and G93A+/− mice. Data are reported as mean ± SEM. ^****^P < 0.0001 (G93A−/− vs G93A+/+; ^###^*P* < 0.001 (G93A−/− vs G93A+/−) by one-way ANOVA with Tukey’s post-analysis. **f** G93A−/− mice spent on average seven days more in the cage after the onset of disability than G93A+/+ mice. ^****^P < 0.0001 (G93A−/− vs G93A+/+); ^##^*P* < 0.01 (G93A−/− vs G93A+/−) by one-way ANOVA with Tukey’s post-analysis. Data are reported as mean ± SEM. All the analysis, except the survival, were performed on *n* = 15 mice NTG+/+; *n* = 15 NTG−/− mice; *n* = 16 G93A+/− mice; *n* = 15 G93A+/+ mice and *n* = 15 G93A−/− mice. The overall survival was calculated on *n* = 22 G93A−/− and *n* = 20 G93A +/+ and *n* = 16 G93A+/− mice


Next, we investigated the effects of MHCI and CTL depletion on motor performance and disease progression in mSOD1 mice and NTG littermates. NTG−/− mice did not show any general health problems or alteration in motor function in comparison with **NTG+/+** mice during the entire duration of the experiment (Fig. 2a). However, G93A−/− mice had earlier onset of paw grip strength impairment about ten days sooner than G93A+/+ mice (Fig. 2b). The age at the onset of muscle weakness was 114.1 ± 6.2 d in G93A−/− mice compared with 121.2 ± 7 d in SOD1^G93A^β2M^+/−^ (**G93A+/−**) and 123.2 ± 7 d in G93A+/+ (*P* < 0.0022 by Mantel-Cox log-rank test) (Fig. 2b). The double genetically modified mice showed no difference in body weight loss compared to G93A+/+ and G93A+/− transgenic mice during disease progression (data not shown). Despite the earlier muscle impairment in G93A−/− mice the disease progressed more slowly than G93A+/+ mice toward complete inability to remain attached to the grid with all four limbs. This time point was defined as the onset of disability and in G93A−/− mice it was 5.8 d later than in G93A+/+ mice (*p* = 0.075) (Fig. 2c). This delay may be attributed to the ability of G93A−/− mice to stay clung longer to the grid with the forelimbs (Additional file 2: Video S1).

**Additional file 2: Video S1.** G93A−/− mice use forelimbs during the last sessions of the PaGE test. An example of a G93A−/− mouse at 154 d mainly using its forelimbs to remain clinging to the grid. (MP4 13626 kb)


Interestingly, G93A−/− mice lived respectively 17 and 10 d longer than G93A+/+ mice and G93A+/− mice (*P* < 0.0001). The G93A+/− mice also survived of 6 d longer than G93A+/+ mice (*P* < 0.015). The average ages (±SD) at death were respectively 164.4 ± 7.2, 171 ± 6.5 and 180.9 ± 10.8 d in the G93A+/+, G93A+/− and G93A−/− mice (Fig. 2d). Thus, the disease in G93A−/− mice lasted significantly longer (64 ± 10.6 d) than in G93A+/+ (42.5 ± 7.3 d, *P* < 0.0001) and in G93A+/− (51.8 ± 9.6 days, *P* < 0.001) mice (Fig. 2e). In agreement with the institutional ethical committee guidelines, the ALS mice must be euthanized when they are unable to right themselves within 10 s after being placed on each side. Given that in mSOD1 mice hindlimbs underwent earlier paralysis, this ability is mainly due to the strength of the forepaws (Additional file 3: Video S2, Additional file 4: Video S3, Additional file 5: Video S4). These results suggest that in G93A−/− mice, while the function of the posterior paws is impaired earlier than in G93A+/+ mice, the preserved function of the forelimbs attenuated the progression of the disease and prolonged the animal ability to stay prone compared to **G93A+/+** mice (Additional file 6: Video S5). Thus, **G93A−/−** mice survived seven and five d more after the onset of disability than **G93A+/+** mice (*P* < 0.0001) and **G93A+/−** mice (*p* < 0.01), respectively (Fig. 2f).


**Additional file 3: Video S2.** SOD1^G93A^ mice at the endstage use forelimbs to pass the survival test. An example of SOD1^G93A^ mice at 168 d using the forelimbs to wrap themselves around in prone position. (MP4 9324 kb)



**Additional file 4: Video S3.** SOD1^G93A^ mice at the endstage use forelimbs to pass the survival test. An example of SOD1^G93A^ mice at 168 d using the forelimbs to wrap themselves around in prone position. (MP4 8287 kb)



**Additional file 5: Video S4.** SOD1^G93A^ mice at the endstage use forelimbs to pass the survival test. An example of SOD1^G93A^ mice at 170 d using the forelimbs to wrap themselves around in prone position. (MP4 6003 kb)



**Additional file 6: Video S5.** G93A−/− mice have better forelimb activity than G93A+/+ mice during the last disease stages. Examples of G93A−/− mice showing good forelimb function on the grid compared to G93A+/+ mice that struggled or could not stay in prone position on the grid, with clear atrophy of at least one anterior paw (Additional file 5: Video S4). (MP4 109814 kb)


We therefore thoroughly investigated the hindlimb neuromuscular system [lumbar spinal cord, sciatic nerves, *Tibialis Anterior* (TA), *Gastrocnemius* (GC)] of G93A+/+ and G93A−/− mice at two time points during the progression of the disease, namely 123 d and 140 d, corresponding respectively to the onset of the hindlimb motor deficit and the advanced symptomatic stage of G93A+/+ mice. Intentionally, G93A+/+ and G93A−/− mice were examined at the same age and not at the same disease stage, to correlate the difference of clinical phenotype with the potential mechanisms involved.

The forepaw neuromuscular system [cervical spinal cord, radial nerves and *Triceps brachii* (TB) muscles] was examined only at the 140 d due to the delayed involvement during the disease course in ALS mice [33, 34].

### The lack of MHCI and CTLs promotes motor neuron survival

**Fig. 3:**
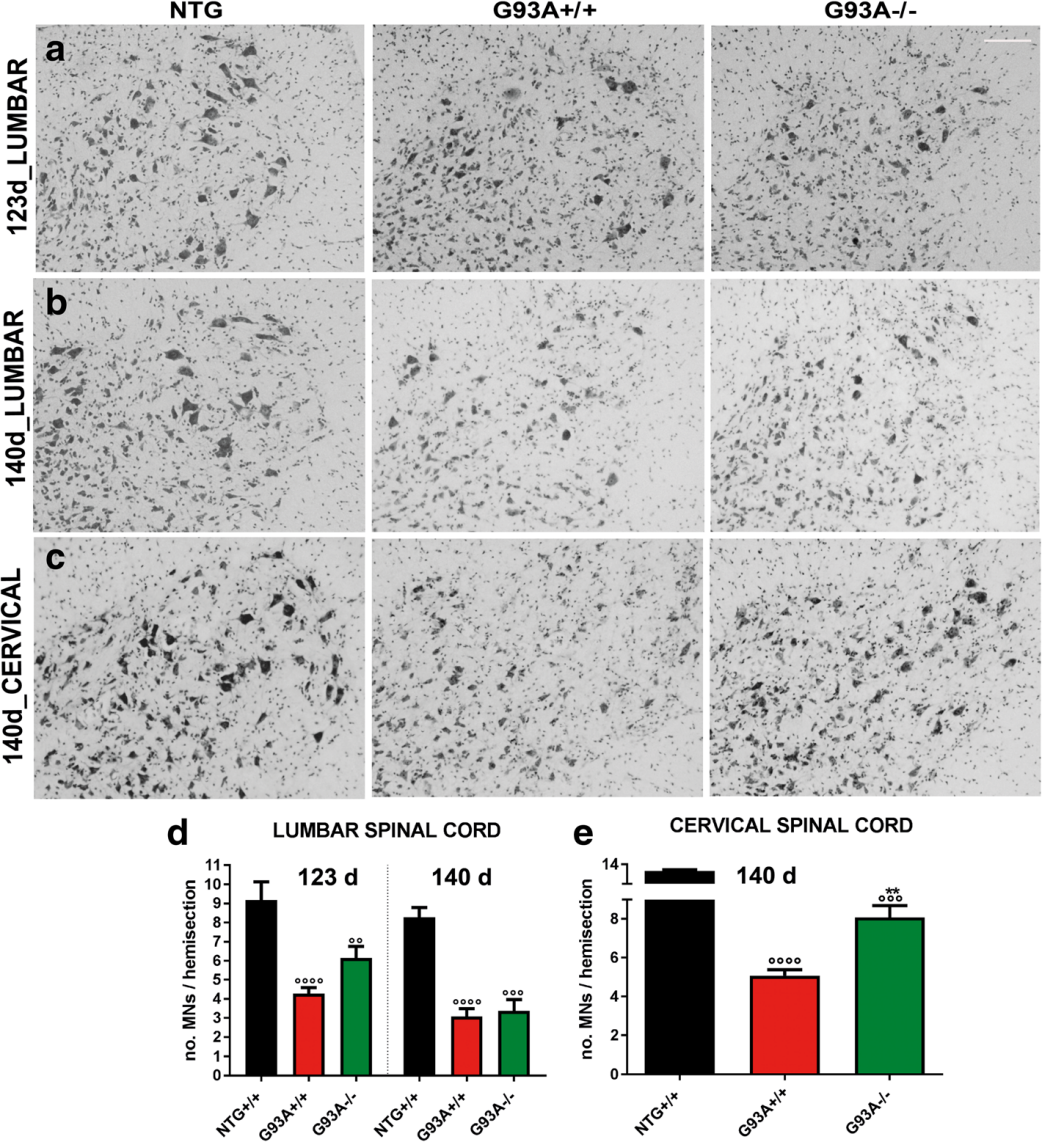
MHCI and CTLs depletion promotes motor neuron survival in mSOD1 mice. **a**, **b** Representative Nissl-stained lumbar spinal cord sections of NTG; G93A+/+ and G93A−/− mice at 123 d. **c** Representative Nissl stained lumbar and cervical spinal cord sections of NTG; G93A+/+ and G93A−/− mice at 140 d. Bar, 50 μm. **d**, **e** Motor neuron counts. Data are expressed as mean ± SEM of MNs (≥ 400 μm^2^) per hemisection. At 123 d, four, seven and seven independent experiments were analyzed for NTG, G93A+/+ and, G93A−/− mice, respectively. At 140 d, three, five and five independent experiments were analyzed for NTG, G93A+/+ and, G93A−/− mice, respectively. At 140 d, for the cervical spinal cord, three, five and five independent experiments were analyzed for NTG, G93A+/+ and, G93A−/− mice, respectively. ^°°°^*P < 0.001;* ^°°°°^ *P < 0.0001* (G93A+/+; G93A−/− vs NTG); ^**^P < 0.001 (G93A−/− vs G93A+/+) by one-way ANOVA with Tukey’s post analysis

We examined whether the more severe hindlimb pathology in G93A−/− mice was related to a higher MN death than in G93A+/+ mice. Large MNs with a cell body area of ≥400 μm^2^ were quantified after Nissl staining in the lumbar spinal cord. Thus only the large α-MNs, the most vulnerable to cell death in ALS, were quantified [35]. Surprisingly, there was a partial, although non-significant, protection of MN in the lumbar spinal cord of G93A−/− compared to G93A+/+ littermates at 123 d but not at 140 d (Fig. 3a, b, d). Moreover, MNs in the cervical spinal cord were significantly protected in G93A−/− mice at 140 d compared to G93A+/+ littermates (Fig. 3c, e). Cervical MNs also preserved their function, as demonstrated by the reduced accumulation of phosphorylated neurofilaments in their perikarya, a marker of neuronal dysfunction and degeneration [24]. In fact, only 4.6% of MNs accumulated SMI-31 (phosphorylated neurofilaments) in their soma in G93A−/− mice, while 23.1% were recordered in G93A+/+ mice (Additional file 1: Figure S2a, b). The same evaluation on the lumbar spinal cord at 123 d did not show a significant difference between G93A+/+ and G93A−/− mice (Additional file 1: Figure S2a, c).

### The lack of MHCI-mediated interaction between microglia and CTLs reduces the inflammation in the spinal cord

**Fig. 4:**
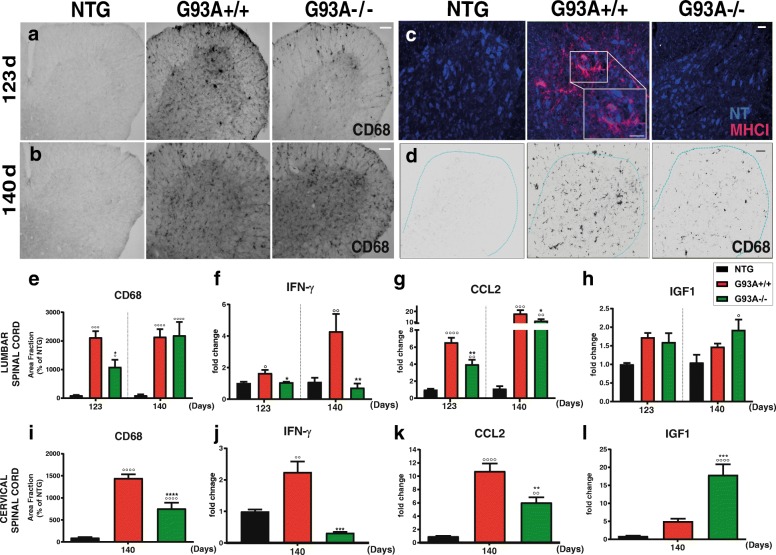
MHCI depletion reduces the inflammation in the lumbar and cervical spinal cord of mSOD1 mice during the disease course. **a**, **b** DAB immunostaining for CD68 in the lumbar spinal cord of NTG; G93A+/+ and G93A−/− mice at 123 and 140 d. Bar, 50 μm. **c** Immunofluorescence staining for MHCI (purple) and motor neurons (neuro-trance, NT; blue) in the cervical spinal cord of NTG; G93A+/+ and G93A−/− mice at 140 d. The inset shows a magnification of MHCI-labeled microglia surrounding MNs. The images are representative of at least four sections from two independent experiments from each genotype. Bar, 50 μm; Inset Bar: 50 μm. **d** Immunofluorescence staining for CD68 in the cervical spinal cord of NTG; G93A+/+ and G93A−/− mice at 140 d. Bar, 50 μm (**e**, **i**) Quantification of CD68 staining in (**e**) lumbar and (**i**) cervical spinal cord hemisections of G93A+/+, G93A−/− mice compared to NTG+/+ littermates at 123 and 140 d. Data are expressed as mean ± SEM (four serial sections for each animal). At 123 d, four, six and four independent experiments were analyzed for NTG, G93A+/+ and, G93A−/− mice, respectively. At 140 d, in cervical and lumbar spinal cord, four independent experiments were analyzed for each genotype. **f-h** Real-time PCR for *Ifn-γ*, *Ccl2* and *Igf1* transcripts in the lumbar spinal cord of G93A+/+, G93A−/− mice compared to NTG+/+ littermates at 123 and 140 d. Data are normalized to β-actin and expressed as the mean ± SEM fold change ratio between G93A+/+ mice, G93A−/− mice and controls from four independent experiments for each genotype. **j-l** Real-time PCR for *Ifn-γ*, *Ccl2* and *Igf1* transcripts in the cervical spinal cord of G93A+/+, G93A−/− mice compared to NTG+/+ littermates at 140 d. Data are normalized to β-actin and expressed as the mean ± SEM fold change ratio between G93A+/+, G93A−/− and controls mice from four independent experiments for each genotype. ^*^P < 0.05; ^**^P < 0.01; ^***^P < 0.001; ^****^P < 0.0001; (G93A+/+ vs G93A−/−); ^°^*P <* 0.05; ^°°^*P < 0.01;* ^°°°^*P < 0.001;* ^°°°°^*P < 0.0001* (G93A−/−; G93A+/+ vs NTG) by one-way ANOVA with Tukey’s post-analysis

We reported that microglia express high levels of MHCI in the lumbar spinal cord of C57SOD1^G93A^ mice [22, 26]. Microglia is the principal antigen-presenting cell and is one of the leading culprits in the non-cell autonomous MN death in ALS [36, 37]. Since induced MHCI in reactive microglia contributes to the activation and recruitment of CD8^+^ T cells [38, 39], we examined wether the lack of interaction between microglia and CD8^+^ T cells reduced inflammation in the CNS of transgenic mice and protected aginst MN loss. At 123 d, the activation of CD68^+^-microglia was lower in the lumbar spinal cord G93A−/− mice than in G93A+/+ littermates. This difference disappeared at 140 d (Fig. 4a, b, e). MHCI-labeled microglia was also observed in the ventral portion of the cervical spinal cord of G93A+/+ mice at 140 d (Fig. 4c). Thus, a significant reduction of CD68^+^-microglia was also observed in the cervical spinal cord of 140 d old G93A−/− compared to G93A+/+ mice (Fig. 4d, i). The decrease of CD68^+^ microglia in both lumbar and cervical spinal cords at respectively 123 d and 140 d was confirmed by the lower levels of CD68 mRNA in G93A−/− mice than in G93A+/+ mice (Additional file 1: Figure S3a, b). No difference was instead found in reactive atrocytosis between G93A−/− mice and G93A+/+ littermates in both lumbar and cervical spinal cord as assessed by GFAP immunohistochemistry and western blot (Additional file 1: Figure S4a-d).

We then examined whether the reduced CD68^+^ microglia activation in both segments of the spinal cord from G93A−/− mice was accompanied by any change in the inflammatory environment compared to G93A +/+ mice, by measuring the expression levels of *Ccl2*, *Ifn-γ, and Igf1* transcripts. We found that the increase of *Ifn-γ* and *Ccl2* in G93A+/+ mice was significantly reduced by the lack of MHCI/CTLs in the lumbar and cervical spinal cord at both 123 and 140 d (Fig. 4f-k). In contrast, *Igf1* was remarkably upregulated in the cervical spinal cord of G93A−/− mice compared to G93A+/+ mice at 140 d while no relevant differences were found in the lumbar spinal cord of mice during the disease progression (Fig. 4h, l). Notably, gliosis and inflammation (see Ccl2; Ifnγ; Igf1mRNA levels) are lower in the cervical than in the lumbar spinal cord of G93A+/+ mice at 140d, further indicating the delayed compromise of the upper versus the lower segment of the spinal cord. This correlates with a lower activation of MHCI by microglia in the cervical compared to lumbar spinal cord (Additional file 1: Figure S5a-c).

To further address the role of microglial MHCI in mediating MN death, we established an in vitro setting composed by cocultures of microglia derived from NTG+/+ or NTG−/− mice added to wild-type (NTG+/+) astrocytes and MNs. These cocultures were exposed to an inflammatory load by 24 h treatment with 1 μg/mL LPS that it is known to induce the MHCI signaling in microglia / macrophages [40, 41].

**Fig. 5:**
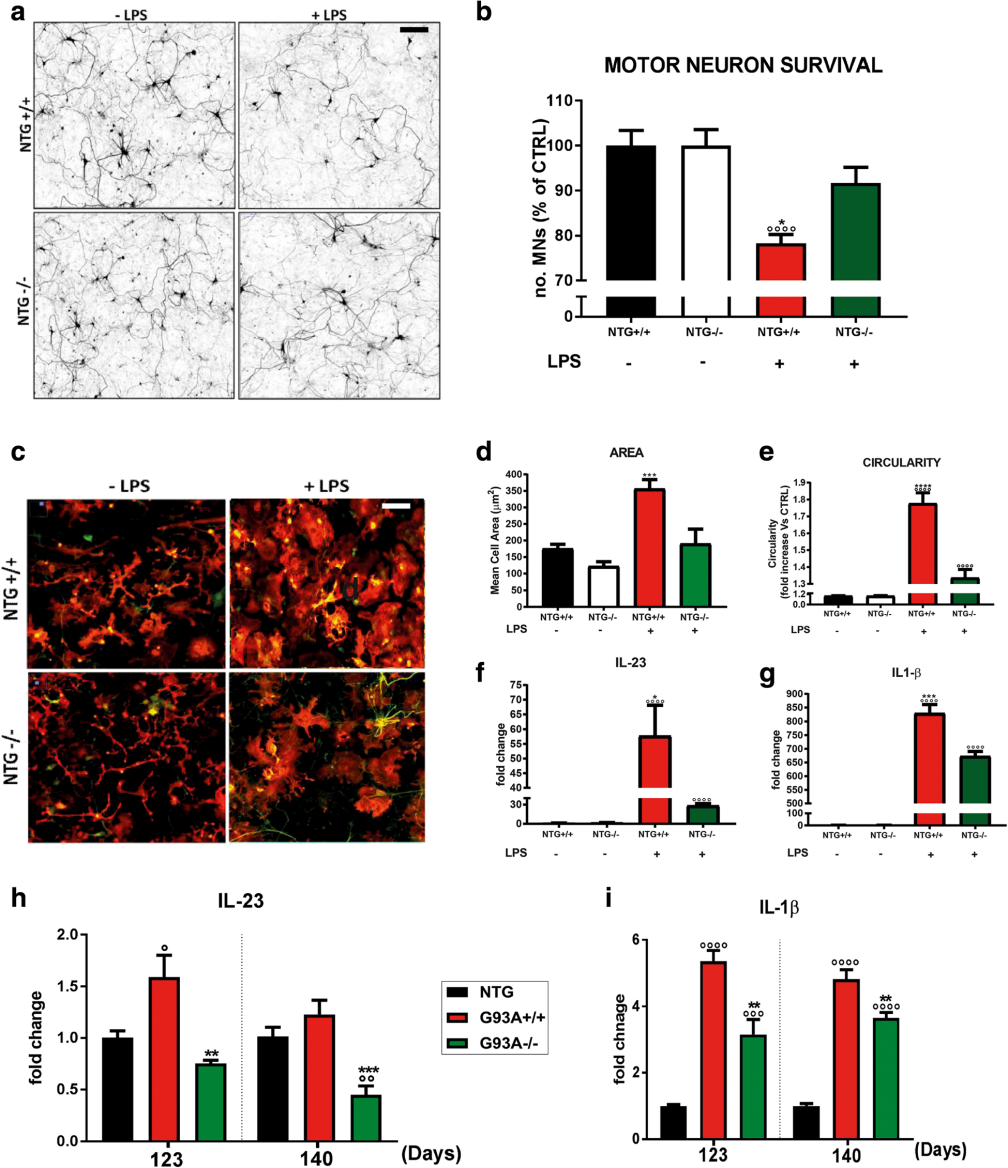
MHCI depletion reduces the activation of primary microglia and motor neuron death in vitro*.* **a** Representative SMI-32 immunostaining images of LPS treated and untreated NTG+/+ motor neurons co-cultured with NTG+/+ or NTG−/− microglia. **b** Cell count analysis showing lower MN loss in LPS treated MNs^NTG+/+^- microglia^NTG−/−^ co-cultures than LPS treated MNs^NTG+/+^- microglia^NTG+/+^ co-cultures.^*^P < 0.05; (LPS_NTG+/+ vs LPS_NTG−/−); ^*°°°°*^*P* < 0.0001 (LPS_NTG+/+; vs NTG+/+; NTG−/−) by one-way ANOVA with Tukey’s post-analysis. **c** Representative IBA-1 immunostaining images (red) of LPS treated and untreated microglia cultures from NTG+/+ and NTG−/− mice. **d**, **e** Morphometric parameters of IBA-1-positive microglia showing lower activation (mean microglial cell area and circularity) of LPS-treated NTG−/− microglia than LPS-treated NTG+/+ microglia. Data are expressed as mean ± SEM from four independent experiments (at least four stereological 0.6 × 0.6 mm fields analyzed per well). **f**, **g** Real-time PCR for *Il-23* and *Il-1β* transcripts from LPS treated and untreated primary microglia cultures from NTG+/+ and NTG−/− mice. Data are normalized to β-actin and expressed as the mean ± SEM fold change ratio between LPS treated NTG+/+ and NTG−/− microglia and relative controls from three independent experiments for each genotype. ^*^P < 0.05; ^***^P < 0.001 (LPS_NTG+/+ vs LPS_NTG−/−); ^*°°°°*^*P* < 0.0001 (LPS_NTG+/+; LPS_NTG−/− vs NTG+/+; NTG−/−) by one-way ANOVA with Tukey’s post-analysis. **h**, **i** Real-time PCR for *Il-23 and Il-1β* transcripts in the lumbar spinal cord of G93A+/+ and G93A−/− mice compared to NTG+/+ littermates at 123 and 140 d. Data are normalized to β-actin and expressed as the mean ± SEM fold change ratio between G93A+/+ mice, G93A−/− mice and control mice from four independent experiments for each genotype. ^*^P < 0.05; ^**^P < 0.01; (G93A+/+ vs G93A−/−); ^*°*^*P <* 0.05; ^*°°°*^*P < 0.001;* ^*°°°°*^*P < 0.0001* (G93A−/−; G93A+/+ vs NTG) by one-way ANOVA with Tukey’s post-analysis

As a result, we observed a reduced LPS-dependent MN death in co-cultures with MHCI depleted microglia (NTG−/−) compared to control NTG+/+ microglia (*p* < 0.05; Fig. 5a, b). Interestingly, after the pro-inflammatory load, microglia from NTG−/− mice showed reduced morphological activation (detected as decreased area and circularity) and lower transcription of *Il-23* and *Il1β* mRNA if compared to NTG+/+ microglia (Fig. 5c-g). qRT-PCR analysis in the lumbar spinal cord of G93A−/− mice showed significant reductions in the transcription of *Il-23* and *Il-1β* compared to G93A+/+ at both 123 and 140 d (Fig. 5h, j). These findings suggest that microglia deprived of MHCI is less sensitive to pro-inflammatory stimuli and become less neurotoxic.

Our data cumulatively point to a shift to an anti-inflammatory environment in the lumbar and cervical spinal cord of G93A−/− mice during the disease course suggesting that the MN preservation, particularly in the cervical spinal cord, is due to a lack of interaction between microglia and CTLs. But, why do G93A −/− mice suffer earlier muscle strength impairment than G93A +/+ mice? To address this, we focused on the peripheral compartment of the MNs: the nerves and neuromuscular junctions.

### Lack of MHCI and CTLs anticipates the denervation atrophy of hindlimb muscles while delaying that of forelimb muscles and diaphragm

Denervation atrophy of muscles is an early event in ALS pathology [42], so we examined whether the more severe motor function impairment in G93A−/− mice was correlated with earlier denervation atrophy of hindlimbs and forelimbs muscles.

**Fig. 6:**
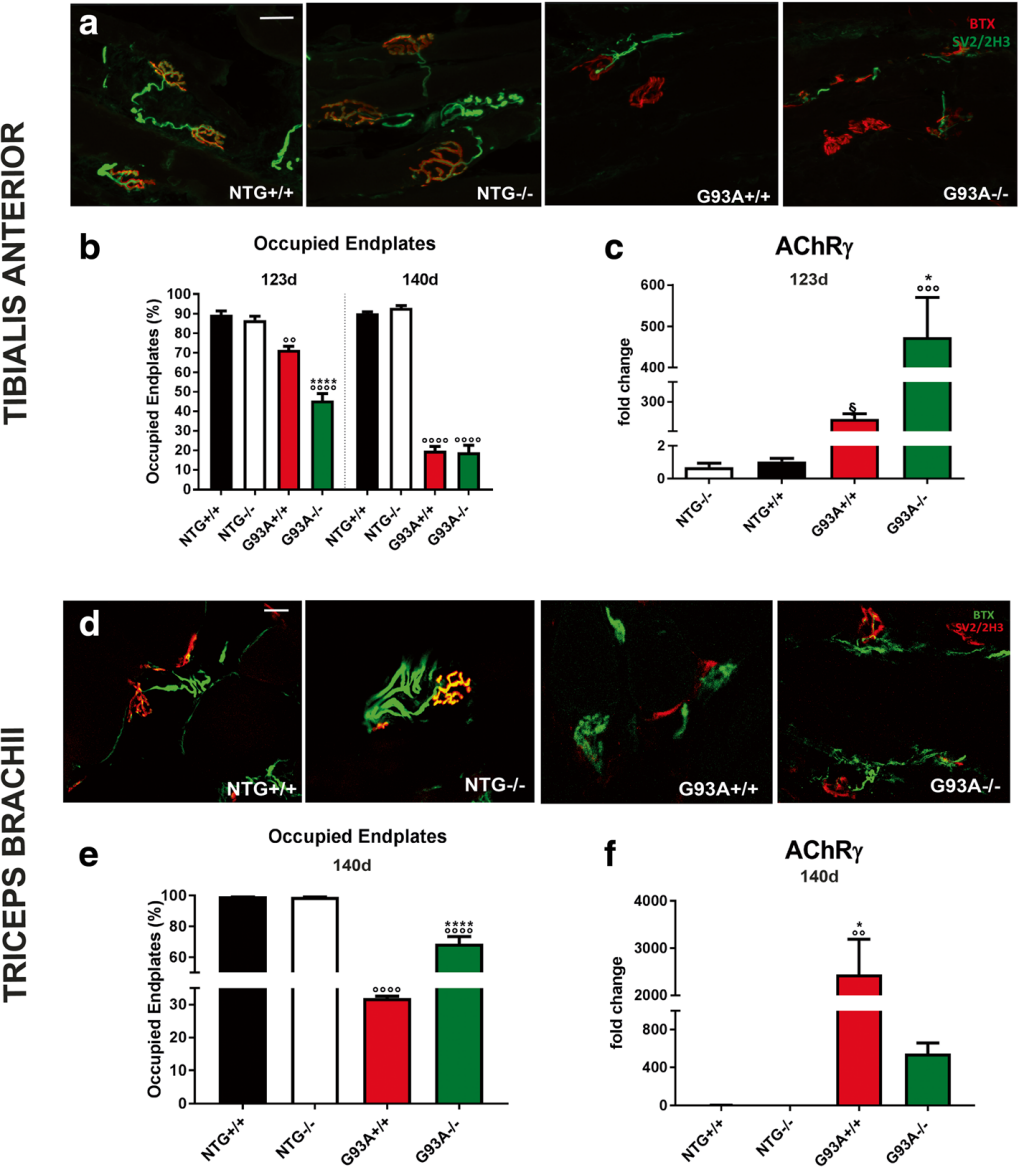
MHCI and CTLs depletion accelerates the denervation of tibialis anterior but delays that of triceps brachii muscle in SOD1 mutant mice. **a** Analysis of muscle denervation on tibialis anterior (TA) muscle of both, G93A+/+, G93A−/− mice and corresponding NTG littermates at 123 d and 140 d. α-Bungarotoxin (BTX, red) was used to identify the postsynaptic domain, synaptic vesicle glycoprotein 2A (SV2, green) + neurofilament (2H3, green) were used to identify presynaptic terminals. Bar, 20 μm. **b** For each mouse group, the percentage of occupied endplates (~ 70 bungarotoxin positive endplates randomly taken) was calculated. Data are reported as mean ± SEM of four independent experiments for each genotype at 123d and from three independent experiments for each genotype at 140 d. ^****^P < 0.0001 (G93A+/+ vs G93A) ^°°^*P* < 0.01; ^°°°°^*P* < 0.001 (G93A+/+ or G93A−/− vs NTG+/+ and NTG−/−) by two-way ANOVA with Sidak’s post-analysis. **c** Real-time PCR for *AChR-γ* transcript in the TA muscles of G93A+/+, G93A−/− mice compared to the corresponding NTG littermates. Data are normalized to β-actin and expressed as the mean ± SEM fold change ratio between G93A+/+ mice, G93A−/− mice and relative controls from four independent experiments for each genotype. ^*^P < 0.05 (G93A−/− vs G93A+/+); ^*°°°*^*P* < 0.001 (G93A−/− vs NTG+/+; NTG−/−); ^§^ *P* < 0.05 (G93A+/+ vs NTG+/+ and NTG−/−). **d** Analysis of muscle denervation on triceps brachii (TB) muscle of G93A+/+ and G93A−/− mice compared to corresponding NTG littermates at 140 d. α-Bungarotoxin (BTX, green) was used to identify the postsynaptic domain, synaptic vesicle glycoprotein 2A (SV2, green) + neurofilament (2H3, red) were used to identify presynaptic terminals. Bar, 20 μm. For each mouse group, the percentage of occupied endplates (~ 70 bungarotoxin positive end plates randomly chosen) was calculated. **e** Data are reported as mean ± SEM. Four, three, three and three independent experiments were analyzed for G93A−/−, G93A+/+, NTG+/+ and NTG−/− mice, mice, respectively. ^****^P < 0.0001; (G93A−/− vs G93A+/+); ^°°°°^*P* < 0.0001 (G93A+/+ or G93A−/− vs NTG+/+ and NTG−/−) by One-way ANOVA with Tukey’s post-analysis. **f** Real-time PCR for *AChR-γ* transcript in the TB muscles of G93A+/+, G93A−/− mice and the corresponding NTG littermates. Data are normalized to β-actin and expressed as the mean ± SEM fold change ratio between G93A+/+, G93A−/− mice and relative controls from four independent experiments for each genotype. ^*^P < 0.05 (G93A−/− vs G93A+/+); ^°°^*P* < 0.001 (G93A+/+ vs NTG+/+; NTG−/−)

**Fig. 7:**
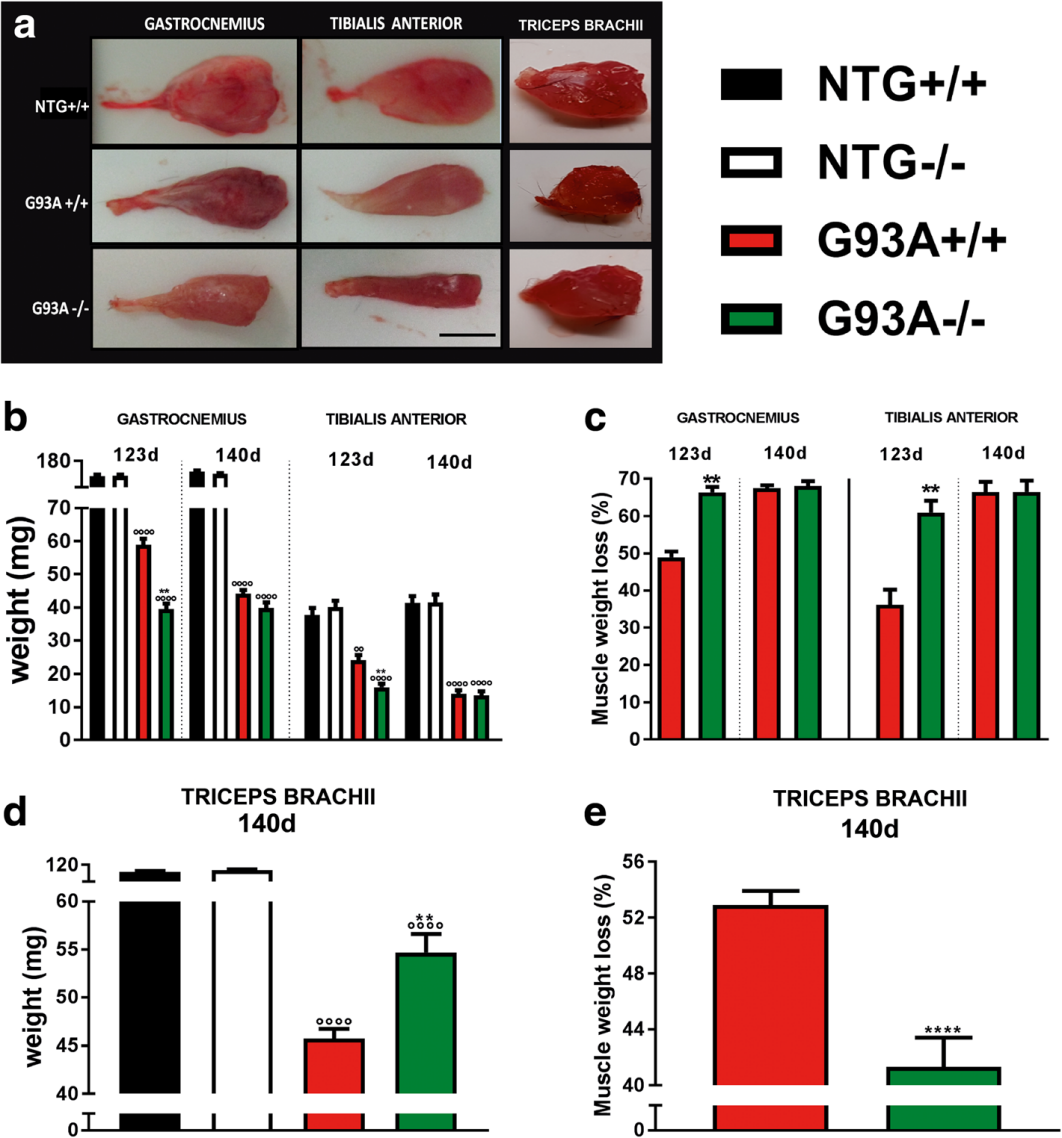
MHCI and CTLs depletion accelerate hindlimb muscle atrophy in SOD1 mutant mice but delays that of triceps brachii muscles in SOD1 mutant mice. (**a**) Representative images of the gastrocnemius, tibialis anterior and triceps brachii muscles showing increased muscle atrophy of hindlimbs muscles in G93A-/- mice at 123 d, but less weight loss of triceps brachii at 140 d compared to G93A+/+ mice; Bar, 0.5 cm. (**b**) Muscle wasting was calculated by measuring of the gastrocnemius and tibialis anterior muscle weight of G93A-/- and G93A+/+ mice compared to relative NTG littermates (NTG+/+; NTG-/-). At 123 d, six, eight, nine and nine GC muscles and six, eight, eight and ten TA muscles were analyzed for NTG+/+, NTG-/-, G93A+/+ and G93A-/- mice, respectively. At 140 d, 11, 14, ten and 16 GC muscles and 11, 14, 16 and 16 TA muscles were analyzed for NTG+/+, NTG-/-, G93A+/+ and G93A-/- mice, respectively. Percent muscle atrophy in (**c**) was calculated relative to NTG mice. (**d**) Triceps brachii muscle wasting was calculated by measurement of the muscle weight of G93A+/+ and G93A-/- mice compared to relative NTG littermates (NTG+/+; NTG-/-) at 140 d. Six, seven, ten and ten independent experiments were analyzed for NTG+/+, NTG-/-, G93A+/+ and G93A-/-, respectively. The percentage of muscle atrophy in (**e**) was calculated relative to corresponding NTG mice. Data are presented as mean ± SEM of three independent experiments for G93A+/+ mice and four independent experiments for G93A-/- mice. ^*^P < 0.05; ^**^P < 0. 01 (G93A+/+ vs G93A-/); ^°°°°^*P* < 0.0001

At 123 d, NMJs in the TA showed more marked denervation in G93A−/− mice with only 45 ± 7.1% remaining innervated compared to 72 ± 3.6 in G93A+/+ mice (Fig. 6a, b). At 140 d, no difference in denervation were observed in the two mSOD1 mice with respectively 18,2 ± 7.4% and 19,8 ± 4.3% of the NMJs remaining innervated in G93A−/− and G93A+/+. At 123 d, the mRNA levels of fetal AChR-γ, a marker of NMJ denervation [43], were significantly more upregulated in G93A−/− than in G93A+/+ TA muscles than in the NTG mice (Fig. 6c). In addition, immunoblot analysis on TA homogenates indicate greater expression of the neuronal AChR-α7 subunit and the neural cell adhesion molecule (NCAM), two markers of disused or denervated muscles [44, 45], in G93A−/− mice than in G93A+/+ mice (Additional file 1: Figure S6a-c). Finally, S100β was markedly higher in the TA of G93A−/− mice at 123 d than in NTG littermates (Additional file 1: Figure S7a, b) suggesting a reduced proliferation of terminal Schwann cells (TSCs) at terminal motor axons [46]. In view of the positive correlation between the number of TSCs and the size of the AChR cluster [47], next we examined the mean AChR cluster area in TA muscles of both mSOD1 mice at 123 d. We identified a specific reduction in the endplate area of G93A−/− mice compared to G93A+/+ and NTG+/+ mice (Additional file 1: Figure S7c, d). Measurements of hindlimb (TA and GC) muscle weight of both transgenic mouse models perfectly reflected the extent of their denervation. At 123 d, G93A−/− mice had greater hindlimb muscle wasting than G93A+/+ mice. G93A−/− mice had weight losses of respectively 66.4 ± 2.5% and 61.4 ± 8% for the GC and TA; G93A +/+ mice had a loss of 48.9 ± 5.3% for the GC and 36.2 ± 2.7% for the TA (Fig. 7a-c). At 140 d, the weight of both TA and GC had fallen further in G93A+/+ mice while in G93A−/− mice it remained unchanged (Fig. 7a-c).

We also confirmed the atrophy of the hindlimbs of G93A−/− mice compared to G93A+/+ mice at 123 d by stereological analysis on transverse sections of TA from mSOD1 mice. First we found a reduction in the mean of muscle fiber cross-sectional area in the TA of G93A−/− mice (Additional file 1: Figure S8a, b). This was reflected in a larger number of fibers with a small diameter (1–1000 μm) and fewer large-diameter fibers (2000–6000 um) (Additional file 1: Figure S8a, c).

To examine the composition of muscle fibers (glycolytic versus oxidative) at 123 d, we employed NADH staining on transverse sections of TA of both transgenic mice groups compared to NTG+/+ mice. This indicted lower density and a smaller percentage of Type IIb fast-fatigable muscle fibers in the hindlimb muscles of G93A−/− (5 ± 2.8%) compared to G93A+/+ (26 ± 5%) mice (Additional file 1: Figure S6d-f). We next looked at the level of innervation of muscles whose activity is directly controlled by the cervical spinal cord. Surprisingly, NMJs in the TB showed the opposite situation to the TA. Denervation was reduced in G93A−/− mice with 68.7 ± 9.2% of the NMJs remaining innervated compared to only 32 ± 1% of G93A+/+ mice (Fig. 6d, e). In keeping with this, the mRNA levels of fetal AChRγ were less upregulated in the TB of G93A−/− mice than in G93A+/+ mice than in NTG mice (Fig. 6f). Besides, in both transgenic mouse models at 140 d there was a weight loss of 41.3 ± 6.5% in G93A−/− mice compared to 52.8 ± 3.2% in G93A+/+ mice (Fig. 7d, e). We also examined the degree of denervation of the diaphragm of both transgenic mice compared to NTG mice. While in G93A+/+ mice the diaphragm innervation was ~ 30% lower, in G93A−/− mice the effect was much smaller with no significant variation with NTG mice (Additional file 1: Figure S9a-c).

### Lack of MHCI and CTLs severely affects the structure of motor axons innervating hindlimb muscles in the course of the disease

Other groups and we have demonstrated that lumbar MNs, after acute injury or chronic disease like in mSOD1 mice activate the expression of MHCI which is rapidly transported into the peripheral axons. Here, it plays a role in the regeneration of motor axons and the stabilization of the NMJs [11, 26, 27, 48]. To clarify the mechanisms underlying this process, we examined the sciatic nerves of G93A−/− and G93A+/+ mice during the progression of disease.

**Fig. 8:**
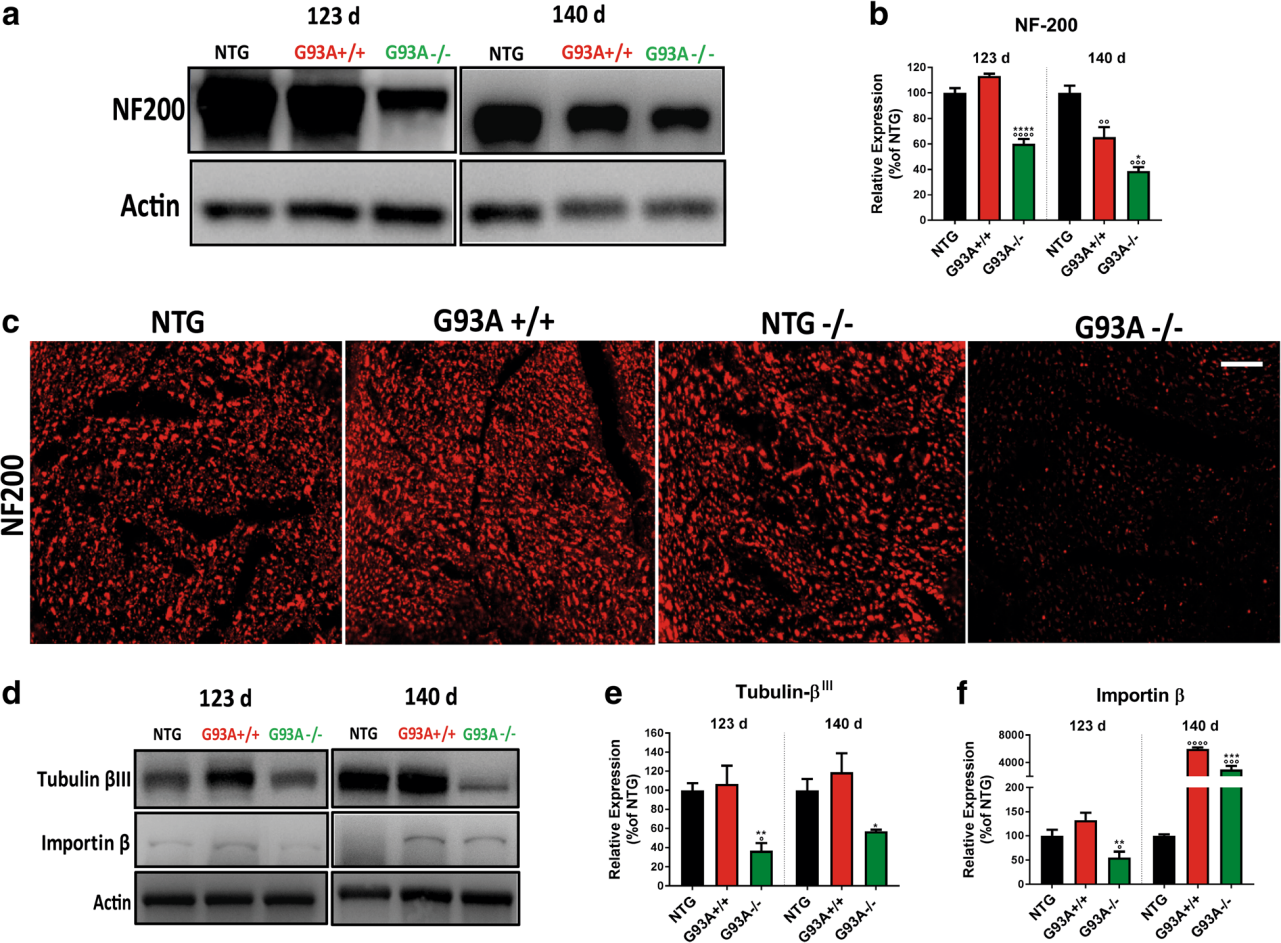
MHCI and CTLs depletion affect the motor axonal cytoskeleton in mSOD1 mice. **a** Representative immunoblot image of neurofilament (NF200) expression in sciatic nerve extracts from NTG, G93A+/+ and G93A^−/−^ mice at 123 and 140 d. **b** Densitometric analysis indicated reductions in the expression levels of neurofilaments in the sciatic nerves of G93A−/− mice during disease progression. At each disease stage, data are reported as percentages of the relative NTG (mean ± SEM) from four independent experiments from each genotype. ^*^P < 0.05; ^**^P < 0.01 (G93A−/− vs G93A+/+); ^*°°*^*P* < 0.01; ^*°°°*^*P* < 0.001; (G93A−/−; G93A+/+ vs NTG+/+) by one-way ANOVA with Tukey’s post-analysis. **c** Confocal micrographs of transverse sections of the sciatic nerve of NTG+/+; NTG−/−,; G93A+/+ and G93A−/− mice, showing markedly lower expression of neurofilaments in G93A−/− mice. The images are representative of at least three sections from two independent experiments for each genotype. Bar, 30 μm. **d** Representative immunoblot images of tubulin-β^III^ and importin β in sciatic nerve extracts from NTG+/+, G93A+/+ and G93A−/− mice at 123 and 140 d. **e**, **f** Densitometric analysis indicated a reduction in the expression levels of (**e**) tubulin-β^III^ and (**f**) importin β in the sciatic nerves of G93A−/− during disease progression. At each disease stage, data are reported as the percentages of NTG (mean ± SEM) from four independent experiments from each genotype. ^*^P < 0.05 ^**^P < 0.01; ^***^P < 0.001 (G93A−/− vs G93A+/+); ^°^*P* < 0.05; ^°°^*P* < 0.001; ^°°°°^*P* < 0.0001 (G93A−/−; G93A+/+ vs NTG) by one-way ANOVA with Tukey’s post-analysis

Immunoblot and immunohistochemical analysis on sciatic nerves showed lower levels of neurofilaments with high molecular weight (200kD) in motor axons (Fig. 8a-c) and a marked reduction in the expression of tubulin β^III^ (Fig. 8d, e) and importin β (Fig. 8d, f) at both 123 d and 140 d in G93A−/− mice compared to G93A+/+ mice. This indicates that the lack of MHCI exacerbate the progressive structural [49, 50] and functional [51, 52] alterations of peripheral motor axons of G93A mice.

**Fig. 9:**
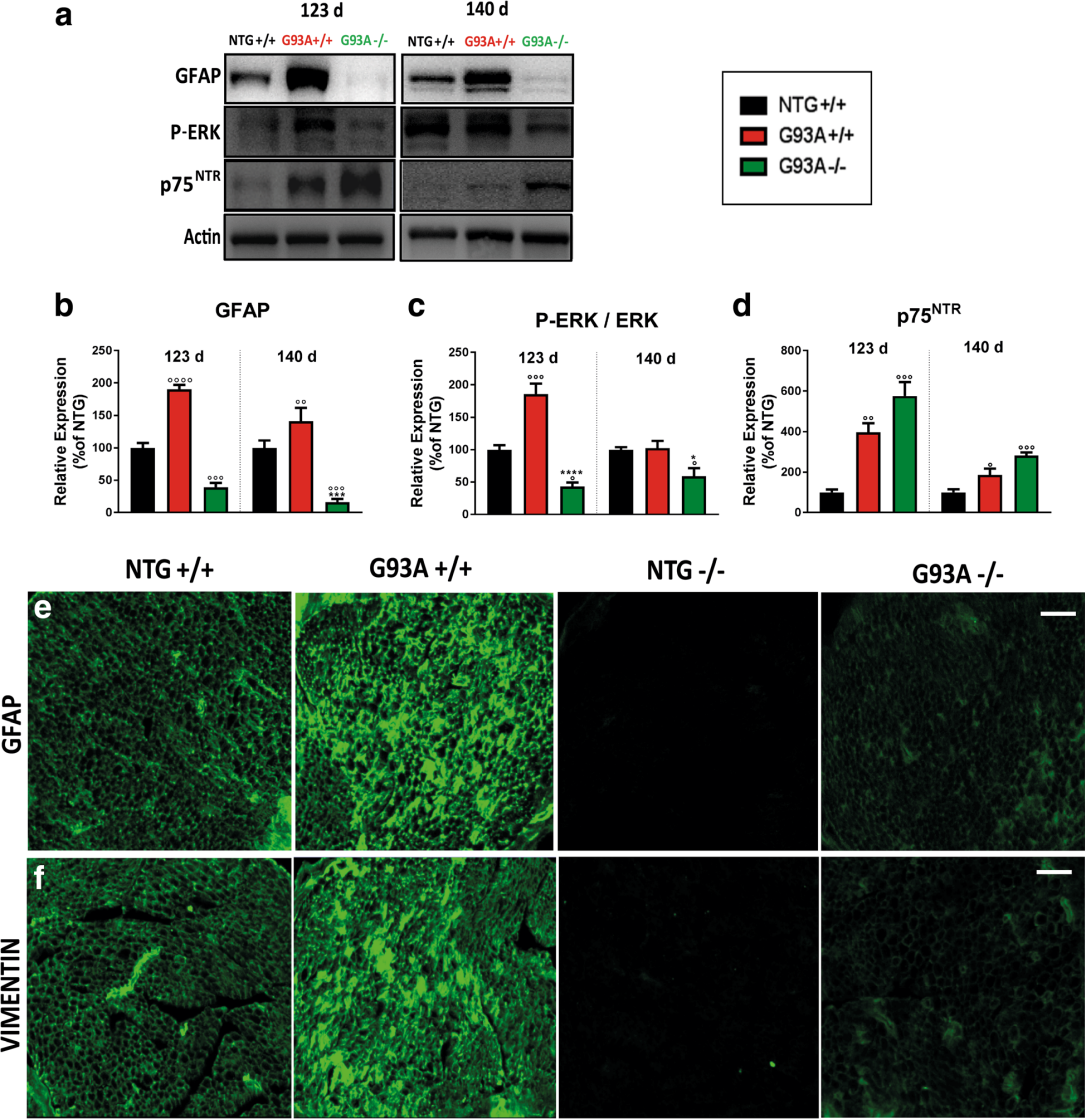
MHCI and CTLs depletion affect the proliferation of Schwann cells in SOD1 mutant mice. **a** Representative immunoblot images of GFAP, phospho-ERK (P-ERK), and p75^NTR^ in sciatic nerve extracts from NTG, G93A+/+ and G93A−/− mice at 123 and 140 d. **b-d** Densitometric analysis indicated a reduction in the expression levels of (**b**) GFAP and (**c**) P-ERK, but (**d**) and increased levels of p75^NTR^ in the sciatic nerves of G93A−/− mice. Relative levels of P-ERK were normalized to levels of total ERK (not shown). At each disease stage, data are reported as the percentage of NTG (mean ± SEM) from four independent experiments from each genotype. ^*^P < 0.05 ^***^P < 0.001; ^****^P < 0.0001 (G93A−/− vs G93A+/+); ^°^*P* < 0.05; ^°°^ *P* < 0.01; ^°°°^*P* < 0.001 (G93A−/−; G93A+/+ vs NTG) by one-way ANOVA with Tukey’s post-analysis. **e-f** Confocal micrographs of transverse sections of sciatic nerve of NTG+/+, NTG−/−; G93A+/+ and G93A−/− mice showing marked reduction in the expression of (**e**) GFAP and (**f**) vimentin in the PNS of NTG−/− and G93A−/− mice at 123 d; The images are representative of at least three sections from two independent experiments for each genotype. Bar, 30 μm

Schwann cells (SCs) are the first-line response to the peripheral damage. They phagocytize the myelin debris and produce the chemotactic signals necessary for correct regeneration [53, 54]. To assess the ability of SCs to respond to stress during ALS progression, we investigated the level of activation of ERK, GFAP, and vimentin in the sciatic nerves of both mSOD mice during the disease progression. These three proteins are essential for the proliferation of the SCs after nerve damage [52–60]. Starting from 123 d, G93A+/+ mice had higher levels of GFAP (Fig. 9a, b, e), the phosphorylated form of ERK (Fig. 9a, c) and vimentin (Fig. 9f) than NTG mice. In contrast, in the sciatic nerves of G93A−/− mice, this response was not present, and the levels of these three markers were even lower than in the NTG mice at both 123 and 140 d. These results suggest substantial impairment in SCs proliferation in G93A−/− mice [59, 60]. However, this does not affect SC de-differentiation as the levels of p75^NTR^ were markedly increased in sciatic nerves of both mSOD1 mice during disease progression (Fig. 6a, d) [53]. Notably, the basal levels of GFAP (Additional file 1: Figure S10a, b); p-ERK (Additional file 1: Figure S10a, c) and vimentin (Fig. 9f) in the sciatic nerves of NTG−/− mice were much lower than in the NTG+/+ mice, indicating that MHCI signaling may have a direct effect on the proliferation of SCs even in the absence of stressful stimuli.

**Fig. 10:**
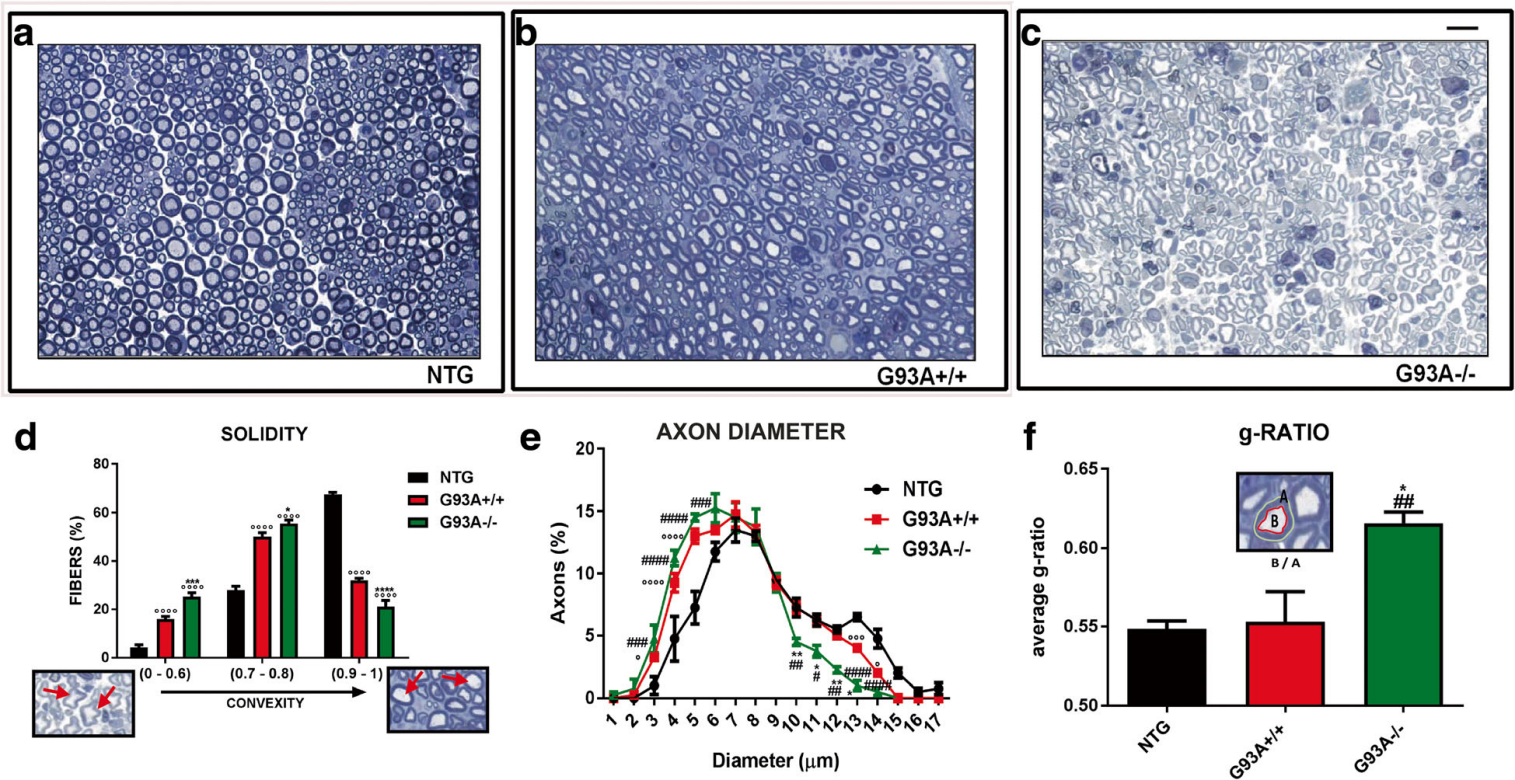
MHCI and CTLs depletion influence the structure and the extent of myelination of motor axons in SOD1 mutant mice (**a**-**c**) Representative images of semithin transverse sections of sciatic nerve from NTG, G93A+/+ and G93A-/- mice at 140 d. Bar, 20 μm. (**d**) Morphometric analysis showing the percentage of distribution of the axonal structure. Arrows in the insets show fibers with an irregular shape (left inset, low convexity) and with a round shape (right inset, high convexity). (**e**) Morphometric analysis showing the percentage of distribution the axonal diameter. (**f**) Average g-ratio in the sciatic nerve from NTG, G93A+/+ and G93A-/- mice. The inset shows the calculation (the ratio between B and A area) to obtain the g-ratio for each axon. Data are reported as mean ± SEM from four independent experiment for each genotype; Data are reported as mean ± SEM from four independent experiments for each genotype. ^°^*P* < 0.05; ^°°^*P* < 0.01; ^°°°^*P* < 0.001; ^°°°°^*P* < 0.0001 (G93A+/+ vs NTG); ^#^*P* < 0.05; ^##^*P* < 0.01; ^###^*P* < 0.001; ^####^*P* < 0.0001 (G93A-/- vs NTG); ^*^P < 0.05; ^**^P < 0.0; ^**^P < 0.001 (G93A-/- vs G93A+/+) by one-way ANOVA with Tukey’s post analysis or unpaired t-test

We next assess the motor axonal structure by a stereological analysis of semithin transverse sections of SNs at the advanced stage of the disease. We first investigated the morphology of the motor axons of G93A−/− and G93A+/+ mice reporting an overall disorganization of the axonal structure in G93A−/− mice (Fig. 10a-c). In fact, there was a larger reduction in the number of rounde fibers (convexity between 0.9 and 1) and a greater increase in the number of fibers with an irregular shape (convexity between 0.0 and 0.6) in sciatic nerves of G93A−/− mice compared to G93A+/+ mice (Fig. 10d). In addition, we identified a significant reduction in the percentage of motor axons with a larger diameter (≥ 10 μm; myelinated axons) in the sciatic nerve of G93A−/− mice in comparison to G93A+/+ mice (Fig. 10e). To evaluate the status of myelination of each nerve fiber we measured the *g-ratio* (ratio between the axon diameter and the fiber diameter) in both G93A+/+ and G93A−/− mice identifyng a specific increase in the sciatic nerves G93A−/− mice (Fig. 10f). In addition, we found lower levels of the four isoforms (21 kD; 18.5 kD; 17.2 kD; 14 kD) of the myelin basic protein (MBP) in thes ciatic nerves of G93A−/− mice compared to G93A+/+ mice (Additional file 1: Figure S11a, b).

### Lack of MHCI and CTLs does not affect the cervical spinal nerves at the advanced disease stage

**Fig. 11:**
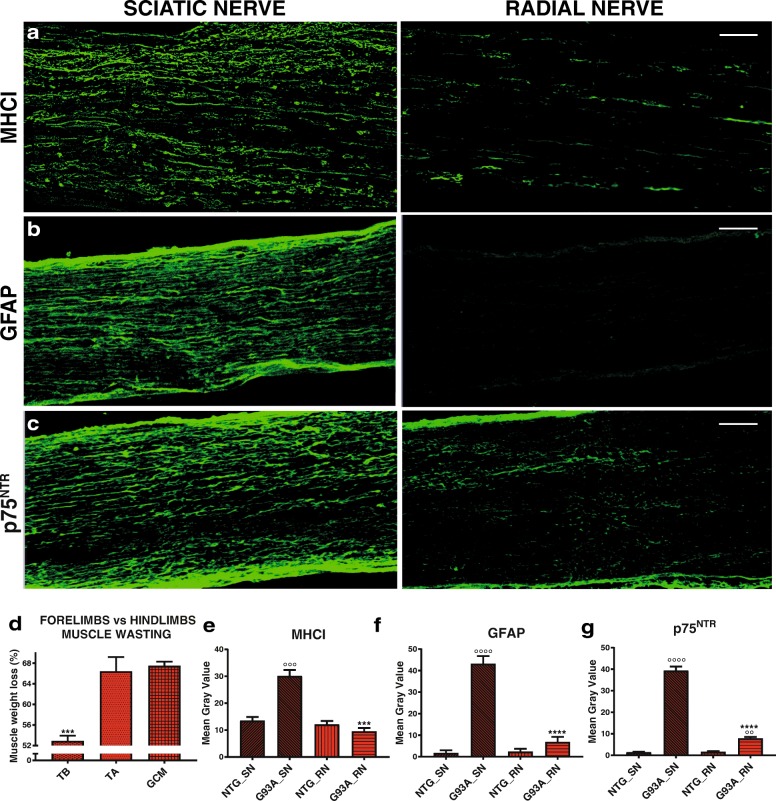
The distal degeneration of forelimbs is slower than that of hindlimbs in SOD1 mutant mice. **a-c** Confocal micrographs of longitudinal sections of the sciatic nerve and radial nerves of G93A+/+ mice at 140 d showing high expression of MHCI, GFAP and p75^NTR^ in the sciatic nerves but not in radial nerves. Bar, 100 μm. **d** Percentages of muscle atrophy (relative to NTG mice) of TB, TA, and GC muscles of G93A+/+ mice at 140 d. Data are expressed as mean ± SEM. Ten, 16 and ten independent experiments were analyzed for TB, TA and GC, respectively. Data are reported as mean ± SEM. ^*^P < 0.05; ^**^P < 0.01; (TA; GC vs TB) by one-way ANOVA with Tukey’s post analysis. **e-g** Quantification of (**e**) MHCI, (**f**) GFAP and (**g**) p75^NTR^ immunoreactivity in sciatic (SN) and radial (RN) nerves of G93A+/+ mice compared to NTG littermates at 140 d. The analysis was done on radial and sciatic nerves of the same animals. Data are expressed as mean ± SEM from three independent experiments (at least four serial sections for each animal) for each genotype. ^***^P < 0.001; ^****^P < 0.0001 (G93A_RN vs G93A_SN) ^°°^*P* < 0.01; ^°°°^*P* < 0.001; ^°°°°^*P* < 0.0001 (G93A_SN or G93A_RN vs NTG_SN or NTG_RN) by one-way ANOVA with Tukey’s post analysis

While MN loss was similar (− 67%) in the cervical and lumbar spinal cord in G93A+/+ mice at the symptomatic stage (140 d), the muscle wasting occurred earlier and more severely in hindlimbs (TA and GC) than forelimbs (TB). At 140 d, the TA and GC of G93A+/+ mice showed weight loss of respectively 67.5 ± 2.6% and 66.3 ± 11.1% compared to 52.8 ± 3.2% in the TB (Fig. 11d). Interestingly, unlike for the sciatic nerves, no or little activation was observed for MHCI (Fig. 11a, e) or stress-related proteins such as GFAP (Fig. 11b, f) and p75^NTR^ (Fig. 11c, g) in radial nerves of G93A+/+ mice at 140 d. However, at the end stage of the disease the levels of all these proteins were significantly increased in the radial nerve even if at a lower extent with respect to the sciatic nerve. (Additional file 1: Figure S12 a-d). This could explain why in mSOD1 mice the disease progression starts from the hindlimbs and only in a second time involves the forepaws. In addition, these data suggest that the activation of MHCI in the periphery is proportional to the degree of damage.

## Discussion

Adaptive immunity, associated with MHCI and infiltrating CTLs, is increasingly recognized as critical in the pathogenesis of many neuroinflammatory diseases, including ALS [5, 22, 26, 27, 61]. Data on CTL infiltration in the damaged area of the brain and spinal cord of ALS patients [10, 17] and mouse models [12, 15] suggest that these cells contribute to MN death. However, the role of the MHCI signlling in the disease pathogenesis is still controversial [5, 26]. Here we provide new information in support of a dual role of the MHCI pathway in the CNS and the PNS over the course of the disease in mSOD1 mice.

We found that the ubiquitary removal of MHCI and depletion of CD8^+^ T cells brought forward the onset of hindlimb force impairment and paralysis in mSOD1 mice due to increased denervation atrophy of hindlimb muscles. In contrast, the forelimb muscles and diaphragm were less denervated in G93A−/− mice, in line with the significant protection of MNs in the cervical spinal cord. This resulted in the prolonged ability of the G93A−/− mouse to bring-back prone with the front paws when placed on its side, despite complete paralysis of the hindlimbs, with a consequent later euthanasia than G93A+/+ mice.

This result contrasts with the study from Staats et al. [62] reporting a shorter survival of β2m−/− SOD1G93A mice compared with the β2m+/− SOD1G93A mice. However, the authors did not observe any increase of CD8 gene expression in the spinal cord of mSOD1 mice suggesting that CTLs did not infiltrate the CNS during the disease progression. We have not explanation for this since there are clear evidence of CD8^+^ T cells infiltration in spinal cord of ALS patients and mSDO1 mice [8, 10, 16, 17].

Furthermore, while we used a large cohort of female mice (according to the standard operating procedures for preclinical animal research in ALS/MND [63]), the number of mice examined in Staats’ work varied between eight and thirteen without indications of gender balancing within the experimental groups although it is well known the sexual dimorphism in the pathology of SOD1G93A mice [64]. All together this evidence may explain the discrepancy of results in the overall survival of β2m−/− SOD1G93A mice.

### MHCI signaling and CD8^+^ T cells infiltration in the PNS enhance the connections of motor axons with hindlimb muscles during the progression of the disease

Our study has strengthened our hypothesis that the specific activation of the MHCI in the sciatic nerves of mSOD1-related ALS mice at disease onset is instrumental in facilitating axonal preservation and maintaining hindlimb muscle innervation, with a positive impact on the early stage of the disease [5, 11, 26]. This partially agrees with Song et al. [27] who recently showed that the specific induction of MHCI in MNs delayed the disease onset and prolong the survival of mSOD1 mice. While they focused mainly on the role of neuronal MHCI overexpression in the CNS in relation to the astrocytes-neuron interaction, little attention was paid to the role of MHCI in the PNS.

We previously showed that 129SvSOD1^G93A^ mice, with faster disease progression and a rapid hindlimb denervation, were unable to activate an MHCI-dependent adaptive immune response in their motor axons, while the slow-progressor C57SOD1^G93A^ mice had a robust increase of MHCI and CTL infiltration in their sciatic nerves [11]. Here we demonstrate that the lack of MHCI activation and CTL infiltration in the PNS of mSOD1 mice destabilizes the peripheral motor axons, which progressively lose their cytoarchitecture and function, exacerbating the denervation of hindlimb muscles. This is accompanied by altered function and proliferation of SCs, preventing the establishment of a favorable environment for collateral re-innervation [53]. To obtain efficient nerve regeneration after damage mature SCs have to dedifferentiate, proliferate, and provide this favorable environment for axonal sprouting [53]. A defect in one of these functions, that imply continuous rearrangement of the cytoskeleton, may result in defective remyelination of motor axons. After axonal damage, both GFAP and Vimentin are upregulated to ensure the efficient cytoskeleton rearrangement necessary for the de-differentiation and proliferation of SCs [65–67]. While GFAP (and the relative activation of ERK) is essential to initiate the proliferation of SCs, Vimentin is involved in sustaining this process until its completion [59]. Accordingly, depletion of GFAP, Vimentin or both delays axonal regeneration and motor recovery after peripheral nerve damage [55, 57, 59, 60]. Here we showed that while G93A+/+ mice strongly activated GFAP and vimentin starting from the disease onset, MHCI depletion affected the basal level of vimentin, GFAP and ERK phosphorylation in the PNS of NTG−/− mice and, as a consequence, their level of activation in pathological conditions. As a result, G93A−/− mice showed a progressive and marked reduction of myelinated fibers in sciatic nerves in addition to a remarked alteration of axonal cytoarchitecture.

These findings suggest that MHCI signaling directly influences the architecture of the sciatic nerve so that MHCI activation in addition to CTLs infiltration in the PNS preserve the quality of connections between motor axons and hindlimb muscles during the disease progression. This scenario resembles that previously reported in experimental mouse models of axon remyelination in which the proliferation and differentiation of precursor cells were accompanied by immune cells infiltration [68]. For example, the depletion or pharmacological inhibition of T-cells following toxin- or virus- induced demyelination leads to an impairment of remyelination [69, 70]. Besides, Bombeiro et al. [71] recently showed that boosting the immune response by early adoptive transfer of activated WT lymphocytes three days after axonal injury improved motor recovery in WT and RAG-KO mice. Overall, these data support the hypothesis that the activation of an immune response in the PNS is essential to promote the targeted destruction of defective motor fibers to create a growth-permissive milieu for sprouting of new neurites [54].

### Distal forelimb pathology is delayed in SOD1G93A mice

Early studies of mSOD1 mice reported that the mice first developed hindlimb tremors, then progressive hindlimb weakness with rapidly deteriorating gait, eventually culminating in paralysis of one or both hindlimbs [34, 72, 73]. Forelimb function remains comparatively spared in ALS mice throughout disease progression [34] indicating a different susceptibility of this motor unit. The delayed forelimb motor weakness in ALS mice was partially explained by Beers et al. [74] showing an augmented protective immune response in the cervical spinal cord. In keeping with this, we found how the gliosis and the overall inflammation (including the extent of MHCI activation by microglia) are attenuated in the cervical spinal cord of G93A+/+ mice compared to the lumbar spinal cord during the disease progression.

Here we also showed that muscle atrophy is more significant in GC and TA than in TB of mSOD1 mice. This is possibly because of different patterns of cellular metabolism and cytoskeletal derangements of the forelimb and hindlimb muscles [75].

We also found that the radial nerves of mSOD1 mice were less susceptible to stress than the sciatic nerves of the same mice. This is in line with Clark et al. [33], showing that at late symptomatic stage (140 d) in forelimbs of mSOD1 mice (with the same backround of the present study), axonal (fragmentation, branching) and NMJ (denervation, fragmentation, and beading) alterations are irrilevant if compared to hindlimb which suggests regional differences in the pathogenic mechanisms underlying the disease. In fact, we did not find any activation of MHCI in radial nerves of mSOD1 mice compared to sciatic nerves at the symptomatic disease stage. However, MHCI is activated at the end-stage of disease with levels similar to those observed in the sciatic nerves at 140 d indicating that the induction of MHCI depends directly on the extent of peripheral stress. In conclusion, data from this work and the literature suggest that the differences in inflammation between the two spinal cord segments of ALS mice, after the initiation of disease, is partially due to the stress related signals that MNs and axons receive from the corresponding skeletal muscle targets. In fact, we previously showed an opposite response to a common early down-regulation of complex I in the two muscles type of SOD1G93A mice with earlier metabolic changes and cytoskeletal derangements of the hindlimbs than forelimbs muscle [75]. This agrees with the evidence that hindlimb muscles are more susceptible to alterations in energy production than forelimb muscles [76].

Therefore we assume that MHCI signaling is essential to preserve the quality of the connections of motor axons with rapidly degenerating hindlimb muscles, independently from MN loss. This could explain the earlier motor onset in G93A−/− mice. In contrast, the denervation atrophy of forelimbs is mainly dependent on the health status of the MN cell body in the cervical spinal cord.

### MHCI activation by microglia and CD8^+^ T cells infiltration in the spinal cord are detrimental to motor neuron survival

The role of the inflammatory response in the PNS stands in stark contrast to that of the CNS, where the reaction of nearby cells is mainly associated with inhibitory scar formation, quiescence, and degeneration/apoptosis [54]. Activated microglia in the CNS can cross-present antigen and stimulate the cytotoxic activity of naive CD8^+^ T cells in a proteasome- and TAP-dependent manner [38, 39]. CD8^+^ T cells progressively infiltrate the spinal cord of SOD1^G93A^ mice [8, 15]. However, the consequences of these events have never been investigated in ALS mice. Here we report that microglia depleted of MHCI are less sensitive to pro-inflammatory stimuli and this, in addition to the lack of CTL infiltration in the CNS, resulted in less inflammation that led to the preservation of MNs in the spinal cord of SOD1^G93A^ mice. In fact, despite the earlier motor onset, G93A−/− mice showed no difference in lumbar MN loss compared to G93A+/+ mice. Moreover, the cervical MNs of these mice were significantly preserved at the advanced stage of the disease in comparison to G93A+/+ mice. These findings comply with our previous evidence showing that rapidly progressing mSOD1 mice had a lower MHCI-dependent adaptive immune response, higher hindlimb muscle denervation but a similar lumbar MN loss than slowing progressing mSOD1mice [11, 28]. Recently, Komine et al. [77] reported that CTLs may not be the main modulator of MHCI-mediated inflammation since their reduction through anti-CD8 antibody did not influence the disease progression of mSOD1 mice. This study lack of a detailed the evaluation of histological signatures (MN loss, inflammation, denervation atrophy) so that we ignore if the inhibition of CTLs infiltration is really ineffective on the disease progression of mSOD1 mice. In fact, we still need to understand why CD8^+^ T cell infiltration within the spinal cord is remarkably elicited in mSOD1 mice. Given that in Komine et al. [77] spinal cord microglia still express MHCI, it is possible that other unknown mechanisms compensate for the reduction of CTLs. Alternatively, the remaining number of CTLs after the inhibition (~ 1000) could be still able to induce a response. Further studies are necessary to disentangle this issue. Nevertheless, our data clearly showed that the lack of MHCI expression by microglia reduces the pro-inflammatory response in vitro and in vivo.

## Conclusions

**Fig. 12:**
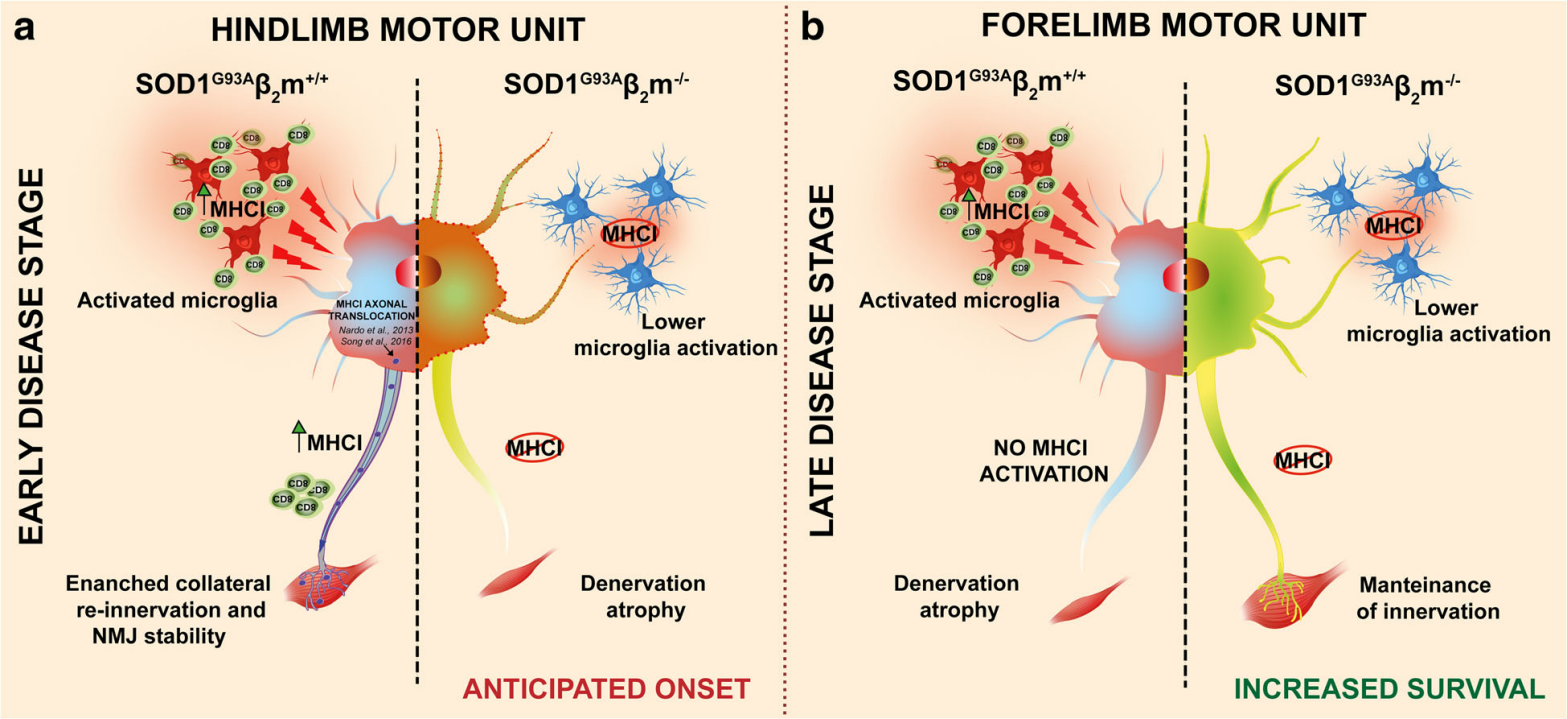
The lack of MHCI signaling anticipated the onset of disease but increase the overall survival of mSOD1 mice. Schematic representation of (**a**) hindlimb and (**b**) forelimb motor unit of SOD1^G93A^B2m^+/+^ (G93A +/+) and SOD1^G93A^B2m^−/−^ (G93A−/−) mice at early and late disease stages. **a** The interaction of MHCI and CTLs in the PNS is essential to preserve the quality of the connections of motor axons with rapidly degenerating hindlimb muscles, independently from MN loss. This could explain the earlier motor onset in G93A−/− mice. These animals, while partially preserving MNs (due the lack of MHCI-mediated interaction of microglia and CTLs), are not able to activate the neuroprotective stress MHCI signalling in the PNS. This lead to an earlier denervation atrophy of hindlimb muscles. **b** In the cervical nerves, the extent of stress is not such as to cause an activation of MHCI signalling in the PNS of G93A+/+ mice. As a consequence, the denervation atrophy of forelimbs is mainly dependent on the health status of the MN cell body in the cervical spinal cord. In G93A−/− mice, the lack of MHCI-mediated interaction of microglia and CTLs results in lower inflammation and higher protection of MNs. Accordingly, the greater functionality of forelimbs causes these mice survive more than G93A+/+ mice

This study illustrates ALS as a complex disease defined by specific pathogenesis in the CNS and PNS and counteracting responses in the lumbar and cervical motor units. We showed that in the lumbar spinal cord motor units of mSOD1 mouse, MN loss is secondary to NMJ destruction and muscle denervation, which are accelerated in the absence of peripheral MHCI activation and CTL infiltration (Fig. 12a). In contrast, in the cervical spinal cord motor units the degree of muscle denervation atrophy is mainly dependent on the viability of MN cell bodies. In this case, the MHCI-dependent interaction between CTLs and microglia plays a crucial role in triggering the neuroinflammation that leads to MN degeneration (Fig. 12b).

Accordingly, a strategy aimed at activating MHCI signaling in the periphery during the early disease stages may be useful to maintaining axonal integrity and maximal connectivity with the muscle, providing a functional reserve for surviving MNs and slowing the disease progression.

In parallel, the inhibition of the MHCI-dependent interaction between CD8^+^ T cells and microglia within the CNS should attenuates the inflammation, prevents MN loss and increases the overall survival.

The failure of non-targeted anti-inflammatory and anti-immune therapies in clinical trials [13, 14] shows up our incomplete knowledge of the dynamic changes that occur during the disease progression and indirectly supports reconsideration of the immune system in ALS. We expect that better understanding of the molecular mechanisms underlying the immune response in transgenic ALS mice should help in finding new approaches for promoting MN survival, axonal regeneration and muscle innervation in ALS patients.

## Additional files

Additional file 1: **Figure S1.** MHCI depletion affect the number of CD3+ / CD4+ T cells but not their extent of infiltration in the spinal cord during the disease progression. **Figure S2.** MHCI depletion reduces the impairment of the cervical motor neurons in mSOD1 mice. **Figure S3.** MHCI depletion lowered the CD68 mRNA levels in the spinal cord of mSOD1 mice. **Figure S4.** MHCI depletion did not affect the extent of astrocytosis in the cervical and the lumbar spinal cord of G93A+/+ mice. **Figure S5.** MHCI expression is lower in the cervical than in the lumbar spinal cord of G93A+/+ mice. **Figure S6.** MHCI depletion accelerates denervation of hindlimb muscles in mSOD1mice. **Figure S7.** MHCI depletion inhibits the proliferation of the terminal Schwann cells and the size of AChR clusters in SOD1 mutant mice. **Figure S8.** MHCI depletion accelerates the atrophy of hindlimbs muscles in SOD1 mutant mice. **Figure S9.** MHCI depletion preserves the diaphragm innervation in SOD1 mutant mice. **Figure S10.** GFAP and phospho-ERK expression are reduced in the sciatic nerve of NTG-/- mice. **Figure S11.** Myelin basic protein isoforms are markedly dwonregulated in the sciatic nerve of G93A-/- mice at 140 d. **Figure S12.** Regional and temporal differences defines the disease progression of mSOD1 mice. (DOCX 21 kb)

## Abbreviations

AChR: Acetyl choline receptor
ALS: Amyotrophic lateral sclerosis
CTLs: Cytotoxic T lymphocites
G93A−/−: SOD1^G93A^β2M^−/−^
G93A+/+: SOD1^G93A^β2M^+/+^
GC: *Gastrocnemius*
MHCI: Major histocompatibility complex I
MN: Motor neuron
NMJ: Neuro muscular junction
NTG: Non transgenic
NTG−/−: Non transgenic β2M^−/−^
NTG+/+: Non transgenic β2M^+/+^
PNS: Peripheral nervous system
SC: Schwann cells
SN: Sciatic nerve
SOD1: Cu/Zn superoxide dismutase
TA: *Tibialis Anterior*
TB: *Triceps Brachii*
TSC: Terminal Schwann cell

## References

[1] Hardiman O, Al-Chalabi A, Chio A, Corr EM, Logroscino G, Robberecht W, Shaw PJ, Simmons Z, van den Berg LH (2017). Amyotrophic lateral sclerosis. Nat Rev Dis Primers.

[2] Zarei S, Carr K, Reiley L, Diaz K, Guerra O, Altamirano PF, Pagani W, Lodin D, Orozco G, Chinea A (2015). A comprehensive review of amyotrophic lateral sclerosis. Surg Neurol Int.

[3] Chia R, Chio A, Traynor BJ (2018). Novel genes associated with amyotrophic lateral sclerosis: diagnostic and clinical implications. Lancet Neurol.

[4] Nardo G, Trolese MC, Tortarolo M, Vallarola A, Freschi M, Pasetto L, Bonetto V, Bendotti C (2016). New insights on the mechanisms of disease course variability in ALS from mutant SOD1 mouse models. Brain Pathol.

[5] Nardo G, Trolese MC, Bendotti C (2016). Major histocompatibility complex I expression by motor neurons and its implication in amyotrophic lateral sclerosis. Front Neurol.

[6] Appel SH, Beers DR, Henkel JS (2010). T cell-microglial dialogue in Parkinson’s disease and amyotrophic lateral sclerosis: are we listening?. Trends Immunol.

[7] Beers DR, Henkel JS, Zhao W, Wang J, Appel SH (2008). CD4+ T cells support glial neuroprotection, slow disease progression, and modify glial morphology in an animal model of inherited ALS. Proc Natl Acad Sci U S A.

[8] Chiu IM, Chen A, Zheng Y, Kosaras B, Tsiftsoglou SA, Vartanian TK, Brown RH, Carroll MC (2008). T lymphocytes potentiate endogenous neuroprotective inflammation in a mouse model of ALS. Proc Natl Acad Sci U S A.

[9] Zhao W, Beers DR, Appel SH (2013). Immune-mediated mechanisms in the pathoprogression of amyotrophic lateral sclerosis. J NeuroImmune Pharmacol.

[10] Holmoy T (2008). T cells in amyotrophic lateral sclerosis. Eur J Neurol.

[11] Nardo G, Trolese MC, de Vito G, Cecchi R, Riva N, Dina G, Heath PR, Quattrini A, Shaw PJ, Piazza V, Bendotti C (2016). Immune response in peripheral axons delays disease progression in SOD1(G93A) mice. J Neuroinflammation.

[12] Chiu IM, Phatnani H, Kuligowski M, Tapia JC, Carrasco MA, Zhang M, Maniatis T, Carroll MC (2009). Activation of innate and humoral immunity in the peripheral nervous system of ALS transgenic mice. Proc Natl Acad Sci U S A.

[13] Khalid SI, Ampie L, Kelly R, Ladha SS, Dardis C (2017). Immune modulation in the treatment of amyotrophic lateral sclerosis: a review of clinical trials. Front Neurol.

[14] McCombe PA, Henderson RD (2011). The role of immune and inflammatory mechanisms in ALS. Curr Mol Med.

[15] NBeers DR, Henkel JS, Zhao W, Wang J, Huang A, Wen S, Liao B, Appel SH (2011). Endogenous regulatory T lymphocytes ameliorate amyotrophic lateral sclerosis in mice and correlate with disease progression in patients with amyotrophic lateral sclerosis. Brain..

[16] Chiu IM, Morimoto ET, Goodarzi H, Liao JT, O’Keeffe S, Phatnani HP, Muratet M, Carroll MC, Levy S, Tavazoie S (2013). A neurodegeneration-specific gene-expression signature of acutely isolated microglia from an amyotrophic lateral sclerosis mouse model. Cell Rep..

[17] Sta M, Sylva-Steenland RM, Casula M, de Jong JM, Troost D, Aronica E, Baas F (2011). Innate and adaptive immunity in amyotrophic lateral sclerosis: evidence of complement activation. Neurobiol Dis.

[18] Peter ME, Budd RC, Desbarats J, Hedrick SM, Hueber AO, Newell MK, Owen LB, Pope RM, Tschopp J, Wajant H (2007). The CD95 receptor: apoptosis revisited. Cell.

[19] Raoul C, Buhler E, Sadeghi C, Jacquier A, Aebischer P, Pettmann B, Henderson CE, Haase G (2006). Chronic activation in presymptomatic amyotrophic lateral sclerosis (ALS) mice of a feedback loop involving Fas, Daxx, and FasL. Proc Natl Acad Sci U S A.

[20] Raoul C, Estevez AG, Nishimune H, Cleveland DW, deLapeyriere O, Henderson CE, Haase G, Pettmann B (2002). Motoneuron death triggered by a specific pathway downstream of Fas. Potentiation by ALS-linked SOD1 mutations. Neuron.

[21] Petri S, Kiaei M, Wille E, Calingasan NY, Flint Beal M (2006). Loss of Fas ligand-function improves survival in G93A-transgenic ALS mice. J Neurol Sci.

[22] Nardo G, Iennaco R, Fusi N, Heath PR, Marino M, Trolese MC, Ferraiuolo L, Lawrence N, Shaw PJ, Bendotti C (2013). Transcriptomic indices of fast and slow disease progression in two mouse models of amyotrophic lateral sclerosis. Brain.

[23] Bendotti C, Marino M, Cheroni C, Fontana E, Crippa V, Poletti A, De Biasi S (2012). Dysfunction of constitutive and inducible ubiquitin-proteasome system in amyotrophic lateral sclerosis: implication for protein aggregation and immune response. Prog Neurobiol.

[24] Cheroni C, Marino M, Tortarolo M, Veglianese P, De Biasi S, Fontana E, Zuccarello LV, Maynard CJ, Dantuma NP, Bendotti C (2009). Functional alterations of the ubiquitin-proteasome system in motor neurons of a mouse model of familial amyotrophic lateral sclerosis. Hum Mol Genet.

[25] Cheroni C, Peviani M, Cascio P, Debiasi S, Monti C, Bendotti C (2005). Accumulation of human SOD1 and ubiquitinated deposits in the spinal cord of SOD1G93A mice during motor neuron disease progression correlates with a decrease of proteasome. Neurobiol Dis.

[26] Chiarotto GB, Nardo G, Trolese MC, França MC Jr, Bendotti C, Rodrigues de Oliveira AL. The Emerging Role of the Major Histocompatibility Complex Class I in Amyotrophic Lateral Sclerosis. Int J Mol Sci. 2017;18(11). 10.3390/ijms18112298 PMC5713268 29104236

[27] Song S, Miranda CJ, Braun L, Meyer K, Frakes AE, Ferraiuolo L, Likhite S, Bevan AK, Foust KD, McConnell MJ (2016). Major histocompatibility complex class I molecules protect motor neurons from astrocyte-induced toxicity in amyotrophic lateral sclerosis. Nat Med.

[28] Marino M, Papa S, Crippa V, Nardo G, Peviani M, Cheroni C, Trolese MC, Lauranzano E, Bonetto V, Poletti A (2015). Differences in protein quality control correlate with phenotype variability in 2 mouse models of familial amyotrophic lateral sclerosis. Neurobiol Aging.

[29] Geuna S, Tos P, Guglielmone R, Battiston B, Giacobini-Robecchi MG (2001). Methodological issues in size estimation of myelinated nerve fibers in peripheral nerves. Anat Embryol (Berl).

[30] De Paola M, Mariani A, Bigini P, Peviani M, Ferrara G, Molteni M, Gemma S, Veglianese P, Castellaneta V, Boldrin V (2012). Neuroprotective effects of toll-like receptor 4 antagonism in spinal cord cultures and in a mouse model of motor neuron degeneration. Mol Med.

[31] Koller BH, Marrack P, Kappler JW, Smithies O (1990). Normal development of mice deficient in beta 2M, MHC class I proteins, and CD8+ T cells. Science.

[32] Zijlstra M, Bix M, Simister NE, Loring JM, Raulet DH, Jaenisch R (1990). Beta 2-microglobulin deficient mice lack CD4-8+ cytolytic T cells. Nature.

[33] Clark JA, Southam KA, Blizzard CA, King AE, Dickson TC (2016). Axonal degeneration, distal collateral branching and neuromuscular junction architecture alterations occur prior to symptom onset in the SOD1(G93A) mouse model of amyotrophic lateral sclerosis. J Chem Neuroanat.

[34] Bruijn LI, Becher MW, Lee MK, Anderson KL, Jenkins NA, Copeland NG, Sisodia SS, Rothstein JD, Borchelt DR, Price DL, Cleveland DW (1997). ALS-linked SOD1 mutant G85R mediates damage to astrocytes and promotes rapidly progressive disease with SOD1-containing inclusions. Neuron.

[35] Friese A, Kaltschmidt JA, Ladle DR, Sigrist M, Jessell TM, Arber S (2009). Gamma and alpha motor neurons distinguished by expression of transcription factor Err3. Proc Natl Acad Sci U S A.

[36] Gerber YN, Sabourin JC, Rabano M, Vivanco M, Perrin FE (2012). Early functional deficit and microglial disturbances in a mouse model of amyotrophic lateral sclerosis. PLoS One.

[37] Weydt P, Yuen EC, Ransom BR, Moller T (2004). Increased cytotoxic potential of microglia from ALS-transgenic mice. Glia.

[38] Jarry U, Jeannin P, Pineau L, Donnou S, Delneste Y, Couez D (2013). Efficiently stimulated adult microglia cross-prime naive CD8+ T cells injected in the brain. Eur J Immunol.

[39] Beauvillain C, Donnou S, Jarry U, Scotet M, Gascan H, Delneste Y, Guermonprez P, Jeannin P, Couez D (2008). Neonatal and adult microglia cross-present exogenous antigens. Glia.

[40] MacAry PA, Lindsay M, Scott MA, Craig JI, Luzio JP, Lehner PJ (2001). Mobilization of MHC class I molecules from late endosomes to the cell surface following activation of CD34-derived human Langerhans cells. Proc Natl Acad Sci U S A.

[41] Rangaraju S, Raza SA, Pennati A, Deng Q, Dammer EB, Duong D, Pennington MW, Tansey MG, Lah JJ, Betarbet R (2017). A systems pharmacology-based approach to identify novel Kv1.3 channel-dependent mechanisms in microglial activation. J Neuroinflammation.

[42] Fischer LR, Culver DG, Tennant P, Davis AA, Wang M, Castellano-Sanchez A, Khan J, Polak MA, Glass JD (2004). Amyotrophic lateral sclerosis is a distal axonopathy: evidence in mice and man. Exp Neurol.

[43] Dobrowolny G, Aucello M, Musaro A (2011). Muscle atrophy induced by SOD1G93A expression does not involve the activation of caspase in the absence of denervation. Skelet Muscle.

[44] Tsuneki H, Salas R, Dani JA (2003). Mouse muscle denervation increases expression of an alpha7 nicotinic receptor with unusual pharmacology. J Physiol.

[45] Covault J, Sanes JR (1985). Neural cell adhesion molecule (N-CAM) accumulates in denervated and paralyzed skeletal muscles. Proc Natl Acad Sci U S A.

[46] Fujiwara S, Hoshikawa S, Ueno T, Hirata M, Saito T, Ikeda T, Kawaguchi H, Nakamura K, Tanaka S, Ogata T (2014). SOX10 transactivates S100B to suppress Schwann cell proliferation and to promote myelination. PLoS One.

[47] Song Y, Panzer JA, Wyatt RM, Balice-Gordon RJ (2006). Formation and plasticity of neuromuscular synaptic connections. Int Anesthesiol Clin.

[48] Thams S, Brodin P, Plantman S, Saxelin R, Karre K, Cullheim S (2009). Classical major histocompatibility complex class I molecules in motoneurons: new actors at the neuromuscular junction. J Neurosci.

[49] Fuller HR, Mandefro B, Shirran SL, Gross AR, Kaus AS, Botting CH, Morris GE, Sareen D (2015). Spinal muscular atrophy patient iPSC-derived motor neurons have reduced expression of proteins important in neuronal development. Front Cell Neurosci.

[50] Lee S, Shea TB (2014). The high molecular weight neurofilament subunit plays an essential role in axonal outgrowth and stabilization. Biol Open.

[51] Perry RB, Doron-Mandel E, Iavnilovitch E, Rishal I, Dagan SY, Tsoory M, Coppola G, McDonald MK, Gomes C, Geschwind DH (2012). Subcellular knockout of importin beta1 perturbs axonal retrograde signaling. Neuron.

[52] Rossi F, Gianola S, Corvetti L (2007). Regulation of intrinsic neuronal properties for axon growth and regeneration. Prog Neurobiol.

[53] Jessen KR, Mirsky R (2016). The repair Schwann cell and its function in regenerating nerves. J Physiol.

[54] Gaudet AD, Popovich PG, Ramer MS (2011). Wallerian degeneration: gaining perspective on inflammatory events after peripheral nerve injury. J Neuroinflammation.

[55] Berg A, Zelano J, Pekna M, Wilhelmsson U, Pekny M, Cullheim S (2013). Axonal regeneration after sciatic nerve lesion is delayed but complete in GFAP- and vimentin-deficient mice. PLoS One.

[56] Tsuda Y, Kanje M, Dahlin LB (2011). Axonal outgrowth is associated with increased ERK 1/2 activation but decreased caspase 3 linked cell death in Schwann cells after immediate nerve repair in rats. BMC Neurosci.

[57] Keller AF, Gravel M, Kriz J (2009). Live imaging of amyotrophic lateral sclerosis pathogenesis: disease onset is characterized by marked induction of GFAP in Schwann cells. Glia.

[58] Agthong S, Kaewsema A, Tanomsridejchai N, Chentanez V (2006). Activation of MAPK ERK in peripheral nerve after injury. BMC Neurosci.

[59] Triolo D, Dina G, Lorenzetti I, Malaguti M, Morana P, Del Carro U, Comi G, Messing A, Quattrini A, Previtali SC (2006). Loss of glial fibrillary acidic protein (GFAP) impairs Schwann cell proliferation and delays nerve regeneration after damage. J Cell Sci.

[60] Perlson E, Hanz S, Ben-Yaakov K, Segal-Ruder Y, Seger R, Fainzilber M (2005). Vimentin-dependent spatial translocation of an activated MAP kinase in injured nerve. Neuron.

[61] Cebrian C, Loike JD, Sulzer D (2014). Neuronal MHC-I expression and its implications in synaptic function, axonal regeneration and Parkinson’s and other brain diseases. Front Neuroanat.

[62] Staats KA, Schonefeldt S, Van Rillaer M, Van Hoecke A, Van Damme P, Robberecht W, Liston A, Van Den Bosch L (2013). Beta-2 microglobulin is important for disease progression in a murine model for amyotrophic lateral sclerosis. Front Cell Neurosci.

[63] Ludolph AC, Bendotti C, Blaugrund E, Chio A, Greensmith L, Loeffler JP, Mead R, Niessen HG, Petri S, Pradat PF (2010). Guidelines for preclinical animal research in ALS/MND: a consensus meeting. Amyotroph Lateral Scler.

[64] McGoldrick P, Joyce PI, Fisher EM, Greensmith L (2013). Rodent models of amyotrophic lateral sclerosis. Biochim Biophys Acta.

[65] Gillen C, Gleichmann M, Spreyer P, Muller HW (1995). Differentially expressed genes after peripheral nerve injury. J Neurosci Res.

[66] Thomson CE, Griffiths IR, McCulloch MC, Kyriakides E, Barrie JA, Montague P (1993). In vitro studies of axonally-regulated Schwann cell genes during Wallerian degeneration. J Neurocytol.

[67] Neuberger TJ, Cornbrooks CJ (1989). Transient modulation of Schwann cell antigens after peripheral nerve transection and subsequent regeneration. J Neurocytol.

[68] Franklin RJ, Kotter MR (2008). The biology of CNS remyelination: the key to therapeutic advances. J Neurol.

[69] Bieber AJ, Kerr S, Rodriguez M (2003). Efficient central nervous system remyelination requires T cells. Ann Neurol.

[70] Begolka WS, Haynes LM, Olson JK, Padilla J, Neville KL, Dal Canto M, Palma J, Kim BS, Miller SD (2001). CD8-deficient SJL mice display enhanced susceptibility to Theiler’s virus infection and increased demyelinating pathology. J Neuro-Oncol.

[71] Bombeiro AL, Santini JC, Thome R, Ferreira ER, Nunes SL, Moreira BM, Bonet IJ, Sartori CR, Verinaud L, Oliveira AL (2016). Enhanced immune response in immunodeficient mice improves peripheral nerve regeneration following axotomy. Front Cell Neurosci.

[72] Gurney ME, Pu H, Chiu AY, Dal Canto MC, Polchow CY, Alexander DD, Caliendo J, Hentati A, Kwon YW, Deng HX (1994). Motor neuron degeneration in mice that express a human cu,Zn superoxide dismutase mutation. Science.

[73] Wong PC, Pardo CA, Borchelt DR, Lee MK, Copeland NG, Jenkins NA, Sisodia SS, Cleveland DW, Price DL (1995). An adverse property of a familial ALS-linked SOD1 mutation causes motor neuron disease characterized by vacuolar degeneration of mitochondria. Neuron.

[74] Beers DR, Zhao W, Liao B, Kano O, Wang J, Huang A, Appel SH, Henkel JS (2011). Neuroinflammation modulates distinct regional and temporal clinical responses in ALS mice. Brain Behav Immun.

[75] Capitanio D, Vasso M, Ratti A, Grignaschi G, Volta M, Moriggi M, Daleno C, Bendotti C, Silani V, Gelfi C (2012). Molecular signatures of amyotrophic lateral sclerosis disease progression in hind and forelimb muscles of an SOD1(G93A) mouse model. Antioxid Redox Signal.

[76] Rosser BW, Norris BJ, Nemeth PM (1992). Metabolic capacity of individual muscle fibers from different anatomic locations. J Histochem Cytochem.

[77] Komine O, Yamashita H, Fujimori-Tonou N, Koike M, Jin S, Moriwaki Y, Endo F, Watanabe S, Uematsu S, Akira S, et al. Innate immune adaptor TRIF deficiency accelerates disease progression of ALS mice with accumulation of aberrantly activated astrocytes. Cell Death Differ. 2018; [Epub ahead of print] 10.1038/s41418-018-0098-3 PMC6261996 29568058

