# Supplementary material for: Counteracting roles of MHCI and CD8+ T cells in the peripheral and central nervous system of ALS SOD1G93A mice
Source: Mol Neurodegener. 2018 Aug 9;13:42. doi: 10.1186/s13024-018-0271-7 (PMC6085701; doi:10.1186/s13024-018-0271-7)
Supplement: Supplementary file 6 — Figure S1. MHCI depletion affect the number of CD3+ / CD4+ T cells but not their extent of infiltration in the spinal cord during the disease progression. Figure S2. MHCI depletion reduces the impairment of the cervical motor neurons in mSOD1 mice. Figure S3. MHCI depletion lowered the CD68 mRNA levels in the spinal cord of mSOD1 mice. Figure S4. MHCI depletion did not affect the extent of astrocytosis in the cervical and the lumbar spinal cord of G93A+/+ mice. Figure S5. MHCI expression is lower in the cervical than in the lumbar spinal cord of G93A+/+ mice. Figure S6. MHCI depletion accelerates denervation of hindlimb muscles in mSOD1mice. Figure S7. MHCI depletion inhibits the proliferation of the terminal Schwann cells and the size of AChR clusters in SOD1 mutant mice. Figure S8. MHCI depletion accelerates the atrophy of hindlimbs muscles in SOD1 mutant mice. Figure S9. MHCI depletion preserves the diaphragm innervation in SOD1 mutant mice. Figure S10. GFAP and phospho-ERK expression are reduced in the sciatic nerve of NTG-/- mice. Figure S11. Myelin basic protein isoforms are markedly dwonregulated in the sciatic nerve of G93A-/- mice at 140 d. Figure S12. Regional and temporal differences defines the disease progression of mSOD1 mice. (DOCX 21 kb) [file 13024_2018_271_MOESM1_ESM.docx]

**Counteracting roles of MHCI and CD8^+^ T cells in the peripheral and central nervous system of ALS SOD1^G93A^ mice**

**Molecular Neurodegeration**

***Giovanni Nardo, Maria Chiara Trolese, Mattia Verderio, Alessandro Mariani, Massimiliano de Paola, Nilo Riva, Giorgia Dina, Nicolò Panini, Eugenio Erba, Angelo Quattrini, Caterina Bendotti***

**Supplementary Figures**

**
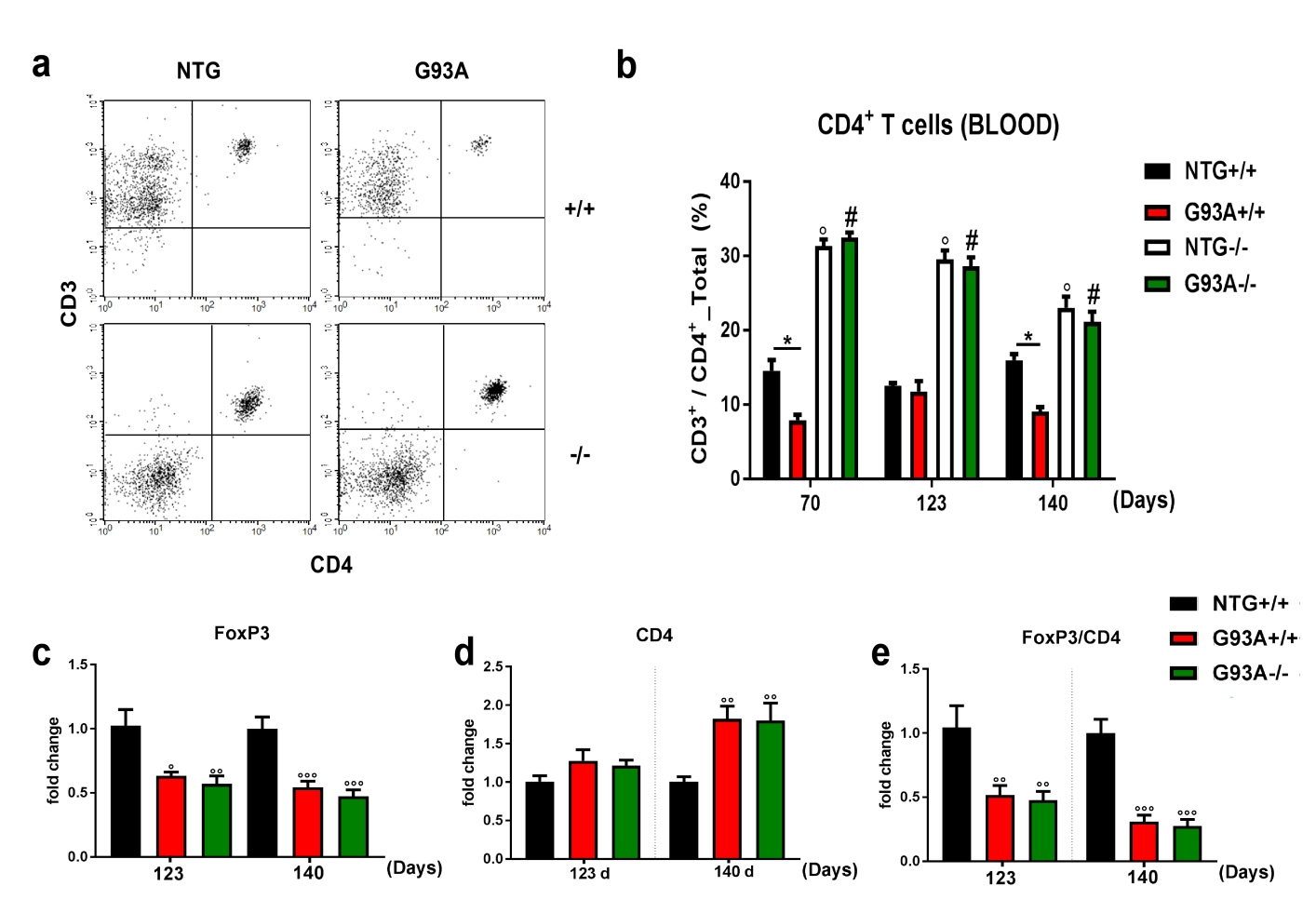
**

**Supplementary Figure 1. MHCI depletion affect the number of CD3^+^ / CD4^+^ T cells but not their extent of infiltration in the spinal cord during the disease progression. (a)** Representative FACS scatter plots of CD3^+^/CD4^+^ T cells in the peripheral blood of NTG+/+ mice, G93A+/+, NTG-/- and G93A-/- mice at 140 d. **(b**) Longitudinal FACS measurement of the percentage of CD3^+^/CD4^+^ T cells in the peripheral blood of G93A+/+; G93A-/- mice and relative controls at 70, 123 and 140 d. Data are reported as the mean ± SEM of six independent experiments (6 mice) for G93A+/+ and NTG+/+ mice and eight independent experiments (8 mice) for G93A-/- and NTG-/- at each time point. *^****^*P *<* 0.0001 (G93A+/+ vs NTG+/+); °°°°P *<* 0.0001 (NTG-/- vs NTG+/+ and G93A+/+); ^####^P *<* 0.0001 (G93A-/- vs NTG+/+ and G93A+/+). (**c, d**) Real-time PCR for the CD4α receptor and FoxP3 transcript in the lumbar and cervical spinal cord of G93A+/+, G93A-/- mice compared to NTG +/+ littermates at 123 and 140 d. Data are normalized to β-actin and expressed as the mean ± SEM fold change ratio between G93A+/+ mice, G93A-/- mice and control mice from four independent experiments for each genotype at both stages. (**e**) FoxP3/CD4 ratio was calculated by dividing the FoxP3 fold change with the CD4 fold change for each mouse. *^**^*P *<* 0.05; *^****^*P *<* 0.0001 (G93A+/+ vs G93A-/-); *^°^*P *< 0.05; ^°°^*P *< 0.01;* (G93A-/- or G93A+/+ vs NTG) by one-way ANOVA with Tukey’s post-analysis.

**
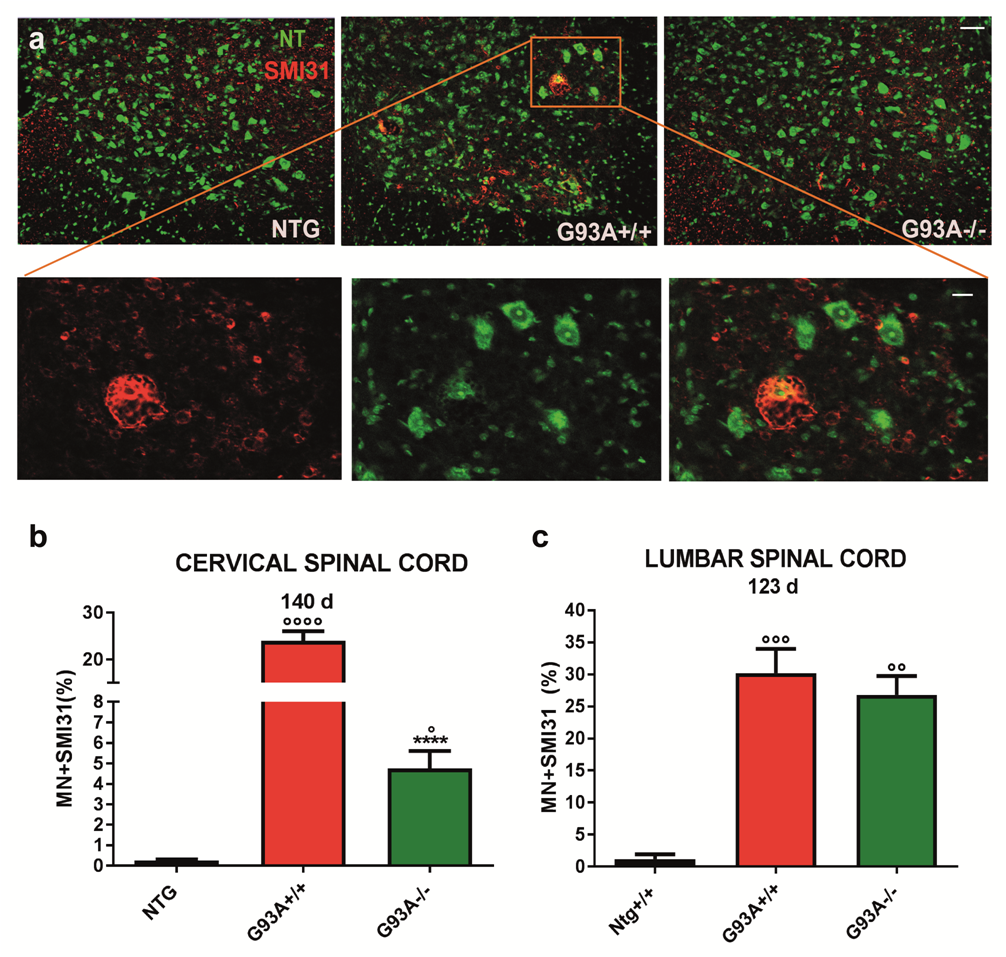
**

**Supplementary Figure 2. MHCI depletion reduces the impairment of the cervical motor neurons in mSOD1 mice.** (**a**) Representative immunofluorescence staining for SMI31 (red) and Neurotrace (NT, green) in the cervical spinal cord of NTG; G93A+/+ and G93A-/- mice. Bar, 50 μm. The inset shows degenerative MN somata positive for SMI31. Bar, 20 μm. (**b-c**) Quantification of the percentages of MNs (≥ 400 μm^2^) positive for SMI31 in the (b) cervical and (c) lumbar spinal cord hemisections of NTG, G93A+/+ and G93A-/- mice at 140 and 123 d, respectively. Data are expressed as mean ± SEM of four independent experiments (from three to six serial sections for each animal) for each genotype. *^****^*P *< 0.0001* (G93A-/- vs G93A+/+)*; ^°°^*P *< 0.01; ^°°°^*P *< 0.001; ^°°°°^*P *< 0.0001* (G93A-/-; G93A+/+ vs NTG); by one-way ANOVA with Tukey’s post-analysis.

**
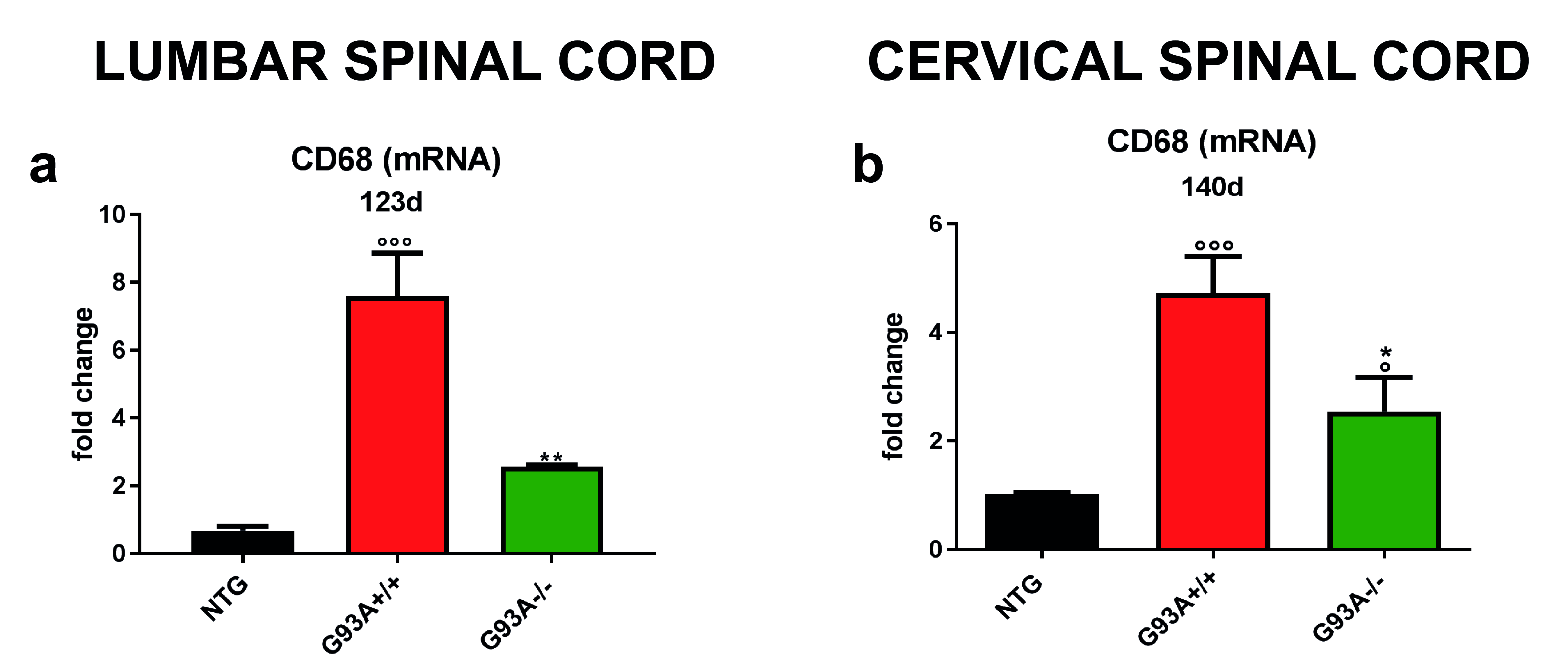
**

**Supplementary Figure 3. MHCI depletion lowered the CD68 mRNA levels in the spinal cord of mSOD1 mice** (**a, b**) Real-time PCR for *CD68* transcript in the (a) lumbar and (b) cervical spinal cords of G93A+/+, G93A-/- mice compared to NTG+/+ littermates at 123 and 140d, respectively. Data are normalized to β-actin and expressed as the mean ± SEM fold change ratio between G93A+/+, G93A-/- mice and control mice from four independent experiments for each genotype. *^*^*P *< 0.05; ^**^*P *< 0.01* (G93A-/- vs G93A+/+); *^°^*P *< 0.05; ^°°°^*P *< 0.001* (G93A-/-; G93A+/+ vs NTG); by one-way ANOVA with Tukey’s post-analysis.

**
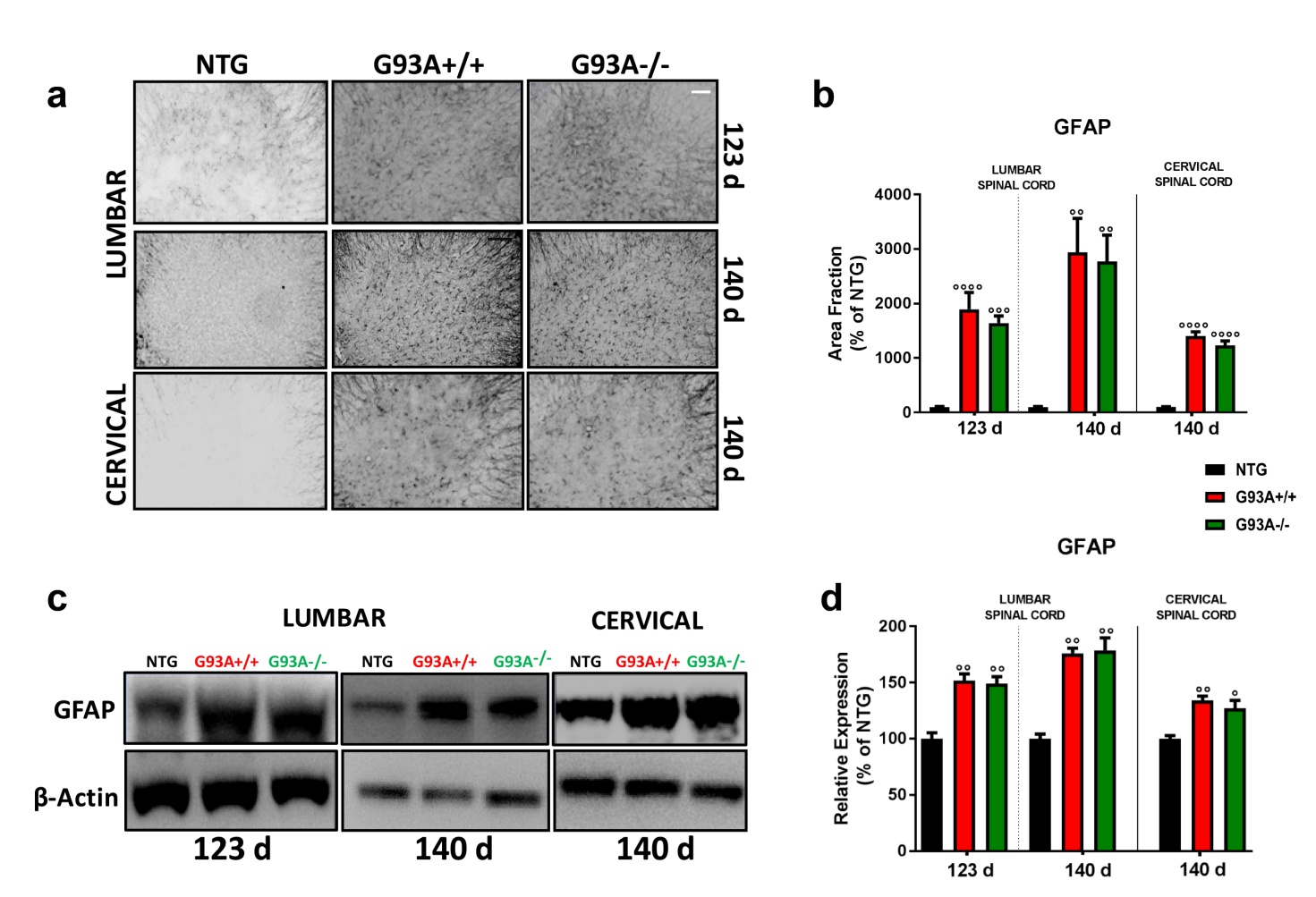
**

**Supplementary Figure 4. MHCI depletion did not affect the extent of astrocytosis in the cervical and the lumbar spinal cord of G93A+/+ mice. (a)** DAB immunostaining for GFAP in the lumbar and cervical spinal cord of NTG; G93A+/+ and G93A-/- mice at 123 and 140 d. The images are representative of at least four sections from four independent experiments from each genotype. Bar, 50 μm; Inset Bar: 50 μm. (**b**) Quantification of GFAP immunostaining in the lumbar and cervical spinal cord of NTG; G93A+/+ and G93A-/- mice at 123 and 140 d. (**c**) Representative immunoblot images of GFAP in the lumbar and cervical spinal cord extracts from NTG+/+, G93A+/+ and G93A-/- mice at 123 d and 140 d. (**d**) Densitometric analysis indicated no difference between G93A+/+ and G93A-/- mice in the extent of activation of GFAP in cervical an lumbar spinal cord at both the time points considered. Data are reported as mean ± SEM from four independent experiments for each genotype. *^°^*P *<* 0.05;  *^°°^*P *< 0.01; ^°°°^*P *< 0.001; ^°°°°^*P *< 0.0001* (G93A-/-; G93A+/+ vs NTG) by one-way ANOVA with Tukey’s post-analysis.

**
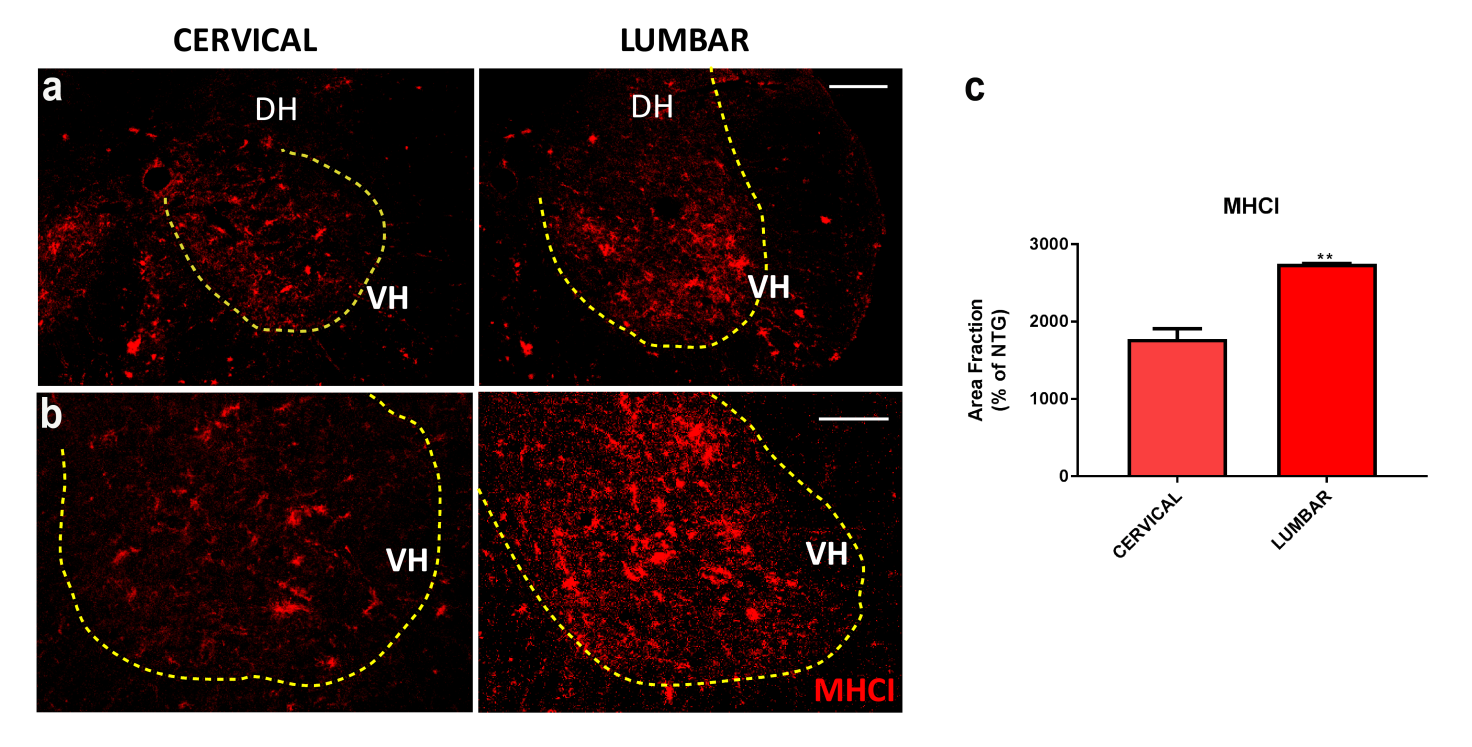
**

**Supplementary Figure 5. MHCI expression is lower in the cervical than in the lumbar spinal cord of G93A+/+ mice. (a)** 10X and (**b**) 20X immunofluorescence staining for MHCI (red) in the cervical and lumbar spinal cord of G93A+/+ mice at 140 d (VH: ventral horn; DH: dorsal horn). (**c**)

Quantification of MHCI immunostaining in the cervical and lumbar spinal cord of G93A+/+ mice at 140 d. The images are representative of at least four sections from three independent experiments. Bar, 50 μm. Data are expressed as percentage of NTG (mean ± SEM). *^**^*P *< 0.01* by t-test.

**
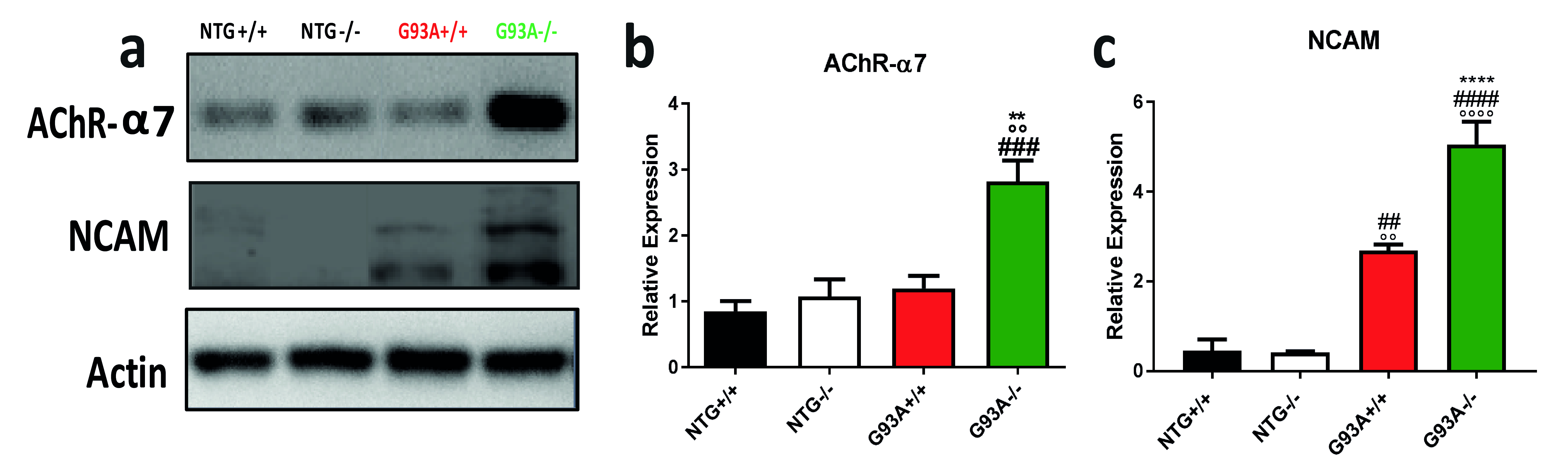
Supplementary Figure 6. MHCI depletion accelerates denervation of hindlimb muscles in mSOD1mice.** (**a**) Representative immunoblot images of AchR-α7 and NCAM in the tibialis anterior muscles extracts from NTG+/+, NTG-/-; G93A+/+ and G93A-/- mice. (**b, c**) Densitometric analysis indicated markedly higher levels of (b) AChR-α7and (c) NCAM in the muscles of G93A-/- mice than in G93A+/+ mice. Data are reported as mean ± SEM from four independent experiments for each genotype. ^**^P *<* 0.01; ^****^P *<* 0.0001 (G93A+/+ vs G93A-/-); *^°°^*P *<* 0.01; *^°°°°^*P *<* 0.0001 (G93A-/-, G93A+/+ vs NTG+/+); *^##^*P *<* 0.01; *^###^*P *<* 0.001; ^#^*^###^*P *<* 0.0001 (G93A-/-; G93A+/+ vs NTG-/-) by one way ANOVA with Tukey’s post-analysis.

**
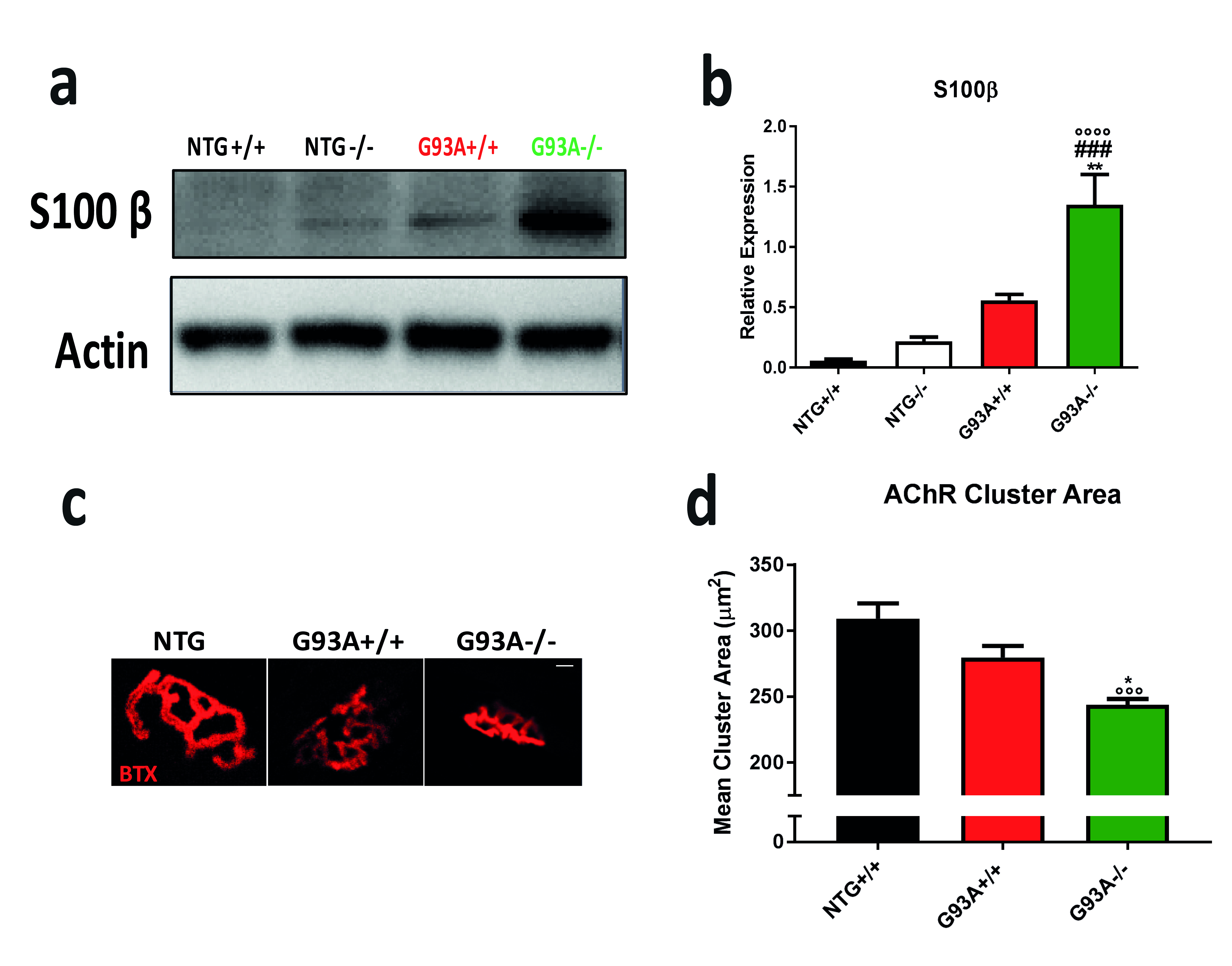
Supplementary Figure 7. MHCI depletion inhibits the proliferation of the terminal Schwann cells and the size of AChR clusters in SOD1 mutant mice.** (**a**) Representative immunoblot images of S100β in the tibialis anterior muscles extracts from NTG+/+; NTG-/-; G93A+/+ and G93A-/- mice. (**b**) Densitometric analysis indicated markedly higher levels of S100β in the muscles of G93A-/- than in G93A+/+ mice. (**c**) Representative images of a single endplate in the TA of NTG+/+; G93A+/+ and G93A-/- mice. Bar, 5 μm. (**d**) Endplate area in the TA of NTG+/+; G93A+/+ and G93A-/- mice (~ 50 bungarotoxin positive end plates were randomly chosen for each mice). Data are reported as mean ± SEM from five independent experiments for each genotype. ^*^P *<* 0.05; ^**^P *<* 0.001 (G93A+/+ vs G93A-/-); *^°°°^*P *<* 0.001; *^°°°°^*P *<* 0.0001 (G93A-/- vs NTG+/+); *^###^*P *<* 0.001; (G93A-/- vs NTG-/-) by one-way ANOVA with Tukey’s post analysis.

**
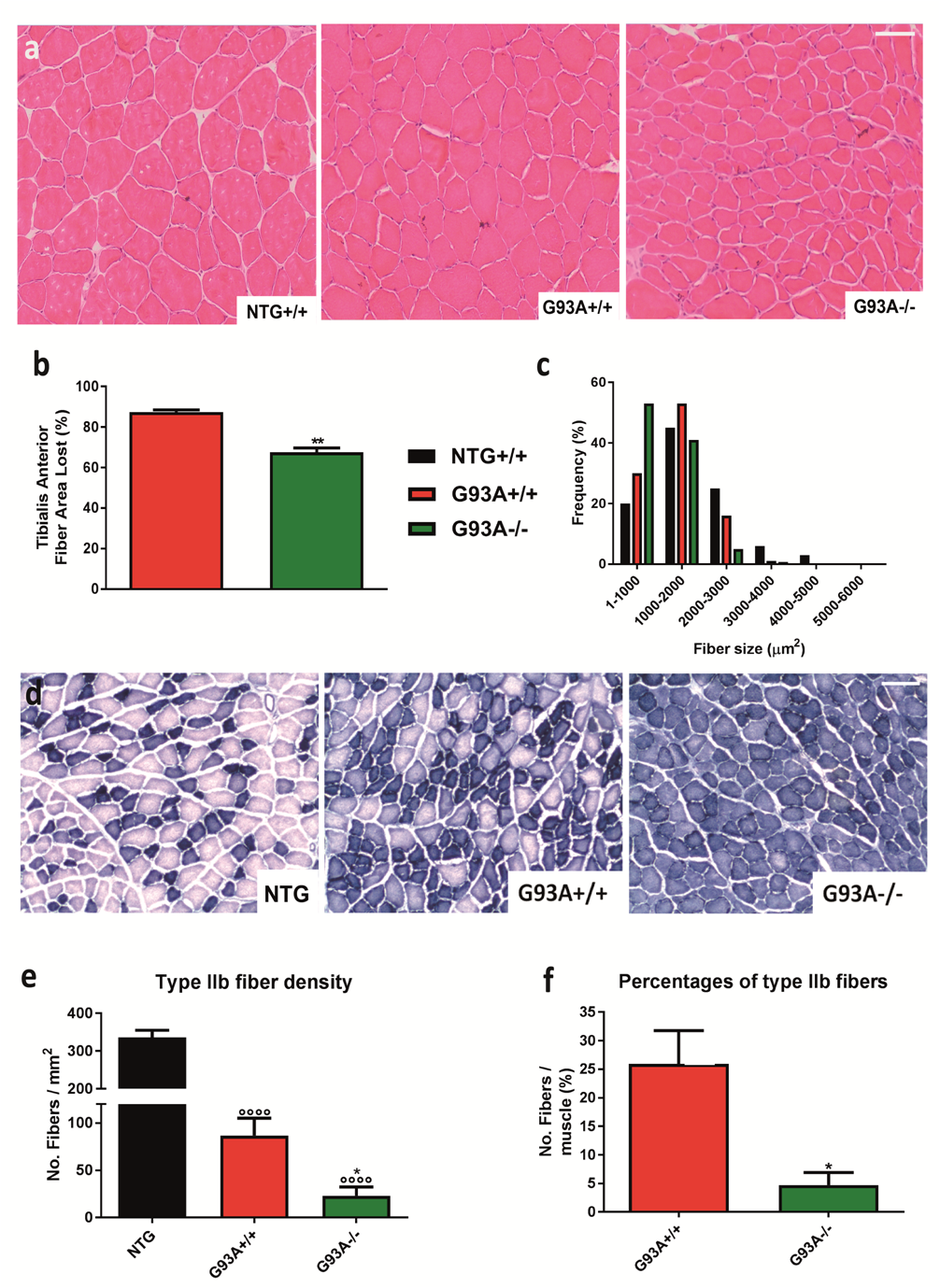
**

**Supplementary Figure 8.** **MHCI depletion accelerates the atrophy of hindlimbs muscles in SOD1 mutant mice**. (**a**) Representative images of a transverse section of tibialis anterior muscle, stained with *H&E*, showing (**b**) a reduction of the mean muscle fiber cross-sectional area (CSA) in G93A-/- mice; Bar, 50 μm. (**c**) Frequency distribution of muscle fibers cross-sectional area of tibialis anterior of G93A-/- and G93A+/+ mice compared to NTG littermates at 140 d. Data are presented as the percentage of the total number of muscle fibers counted from three independent experiments for NTG and G93A+/+ mice and four independent experiments for G93A-/- mice. (**d**) Representative image of a transverse section of the tibialis anterior muscles, stained with NADH, showing a greater reduction of Type II fibers (*white*) in G93A-/- mice. Bar, 50 μm. (**e**, **f**) Density and percentage of type IIb fibers in the tibialis anterior muscles of G93A-/- and G93A+/+ mice compared to NTG littermates. Percentage of fibers was calculated relative to NTG mice. Data are presented as mean ± SEM from four independent experiments for NTG and G93A+/+ mice and five independent experiments for G93A-/- mice. (E): *^*^*P *<* 0.05; *^**^P <* 0.01; (G93A+/+ vs G93A-/-); *^°°°°^*P *<* 0.0001 (G93A-/-; G93A+/+ vs NTG); by one-way ANOVA with Tukey’s post-analysis. (F) *^*^*P *< 0.05*; *^**^*P *< 0.01* by unpaired t-test**.**

**
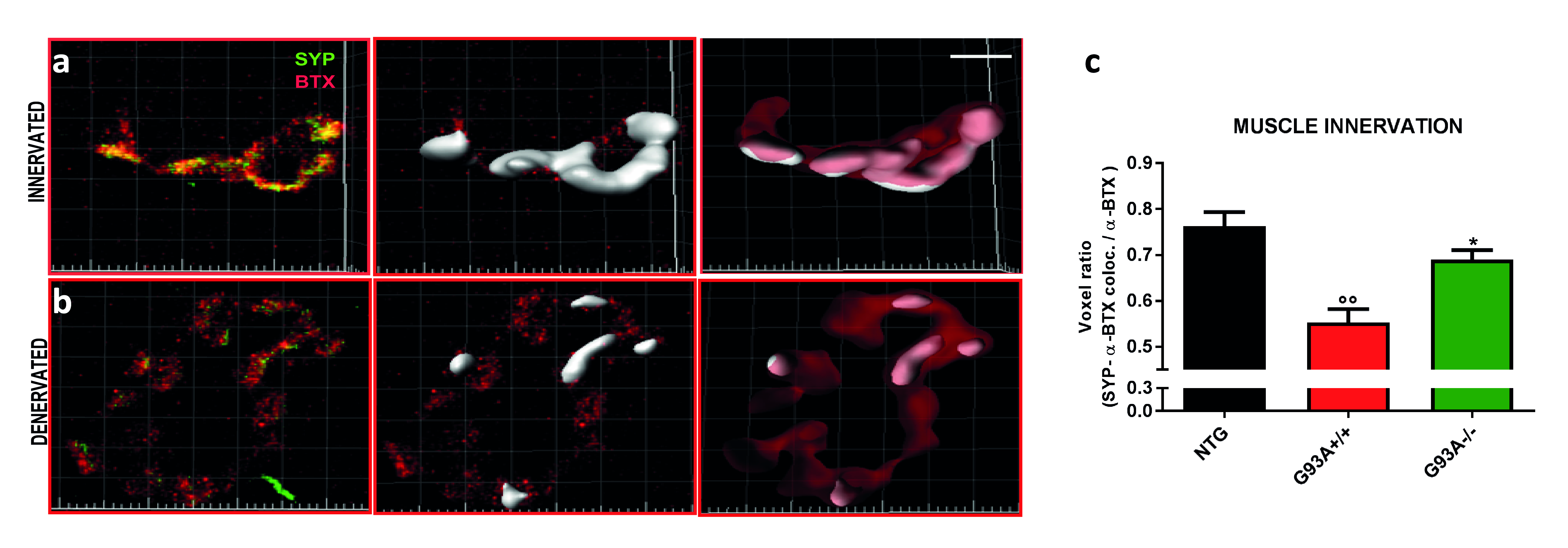
**

**Supplementary Figure 9. MHCI depletion preserves the diaphragm innervation in SOD1 mutant mice** (**a, b**) Representative surpass views and 3D rendering of (a) innervated and (b) denervated endplates in the diaphragm of a G93A+/+ mouse. For each image, the channel (gray) generated from the juxtaposition between synaptophysin (SYP, green) and BTX (red) was converted to isosurface volume and related to the isosurface volume (in voxels) of the BTX channel. Bar, 5 μm (**c**) Quantification of SYP/BTX colocalization / BTX voxels ratio in the diaphragm of NTG, G93A+/+ and G93A-/- mice at 140 d. Data are expressed as mean ± SEM of four independent experiments (at least four serial sections for each animal) for each genotype. *^*^*P *< 0.05* (G93A-/- vs G93A+/+); *^°°^*P *< 0.01;* (G93A+/+ vs NTG).

**
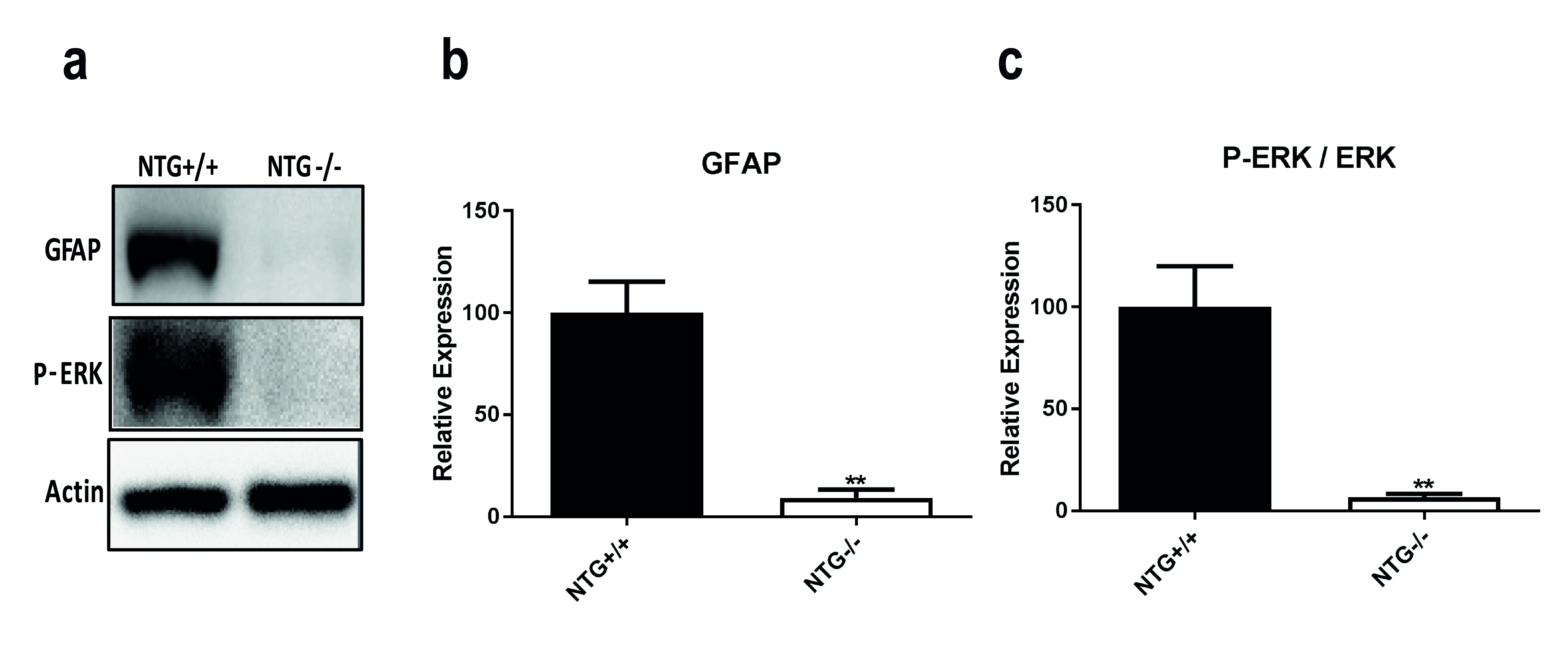
Supplementary Figure 10. GFAP and phospho-ERK expression are reduced in the sciatic nerve of NTG-/- mice.** (**a**-**c**) Representative immunoblot images of phospho-ERK (P-ERK) and GFAP in the sciatic nerve extracts from NTG+/+ and NTG-/- mice at 123 d. Densitometric analysis indicated a marked lower levels of GFAP and P-ERK in age-matched NTG-/- mice. Relative levels of P-ERK were normalized to levels of total ERK (not shown). Data are reported as mean ± SEM of four independent experiments for each genotype. ^**^P < *0.01* by unpaired t-test.


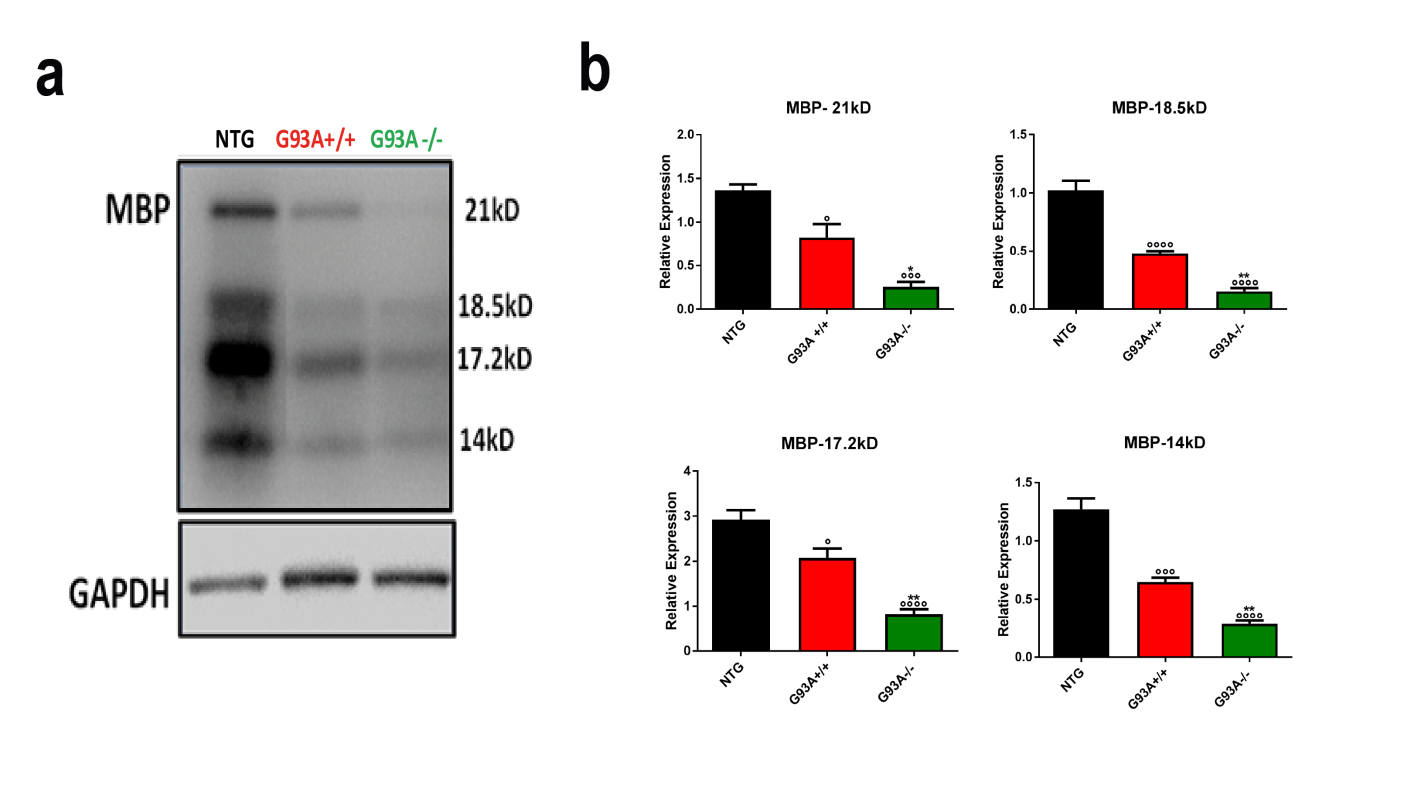
**Supplementary Figure 11. Myelin basic protein isoforms are markedly dwonregulated in the sciatic nerve of G93A-/- mice at 140 d.** (**a)** Representative immunoblot images of the four isoforms of MBP (21kD; 18.5kD; 17.2 kD and 14 kD) in sciatic nerve extracts from Ntg+/+, G93A+/+ and G93A-/- mice at 140 d. (**b**) Densitometric analysis revealed a remarkable reduction of MBP isoforms in G93A-/- in respect to G93A+/+. Data are reported as mean ± SEM from five and four independent experiments for NTG and G93A+/+, G93A-/-, respectively. ^°°^P < 0.001; ^°°°^P < 0.001; ^°°°°^P < 0.0001 (G93A+/+; G93A-/- vs NTG); ^*^P < 0.05; ^**^P < 0.01 by one-way ANOVA with Tukey’s post analysis.

**
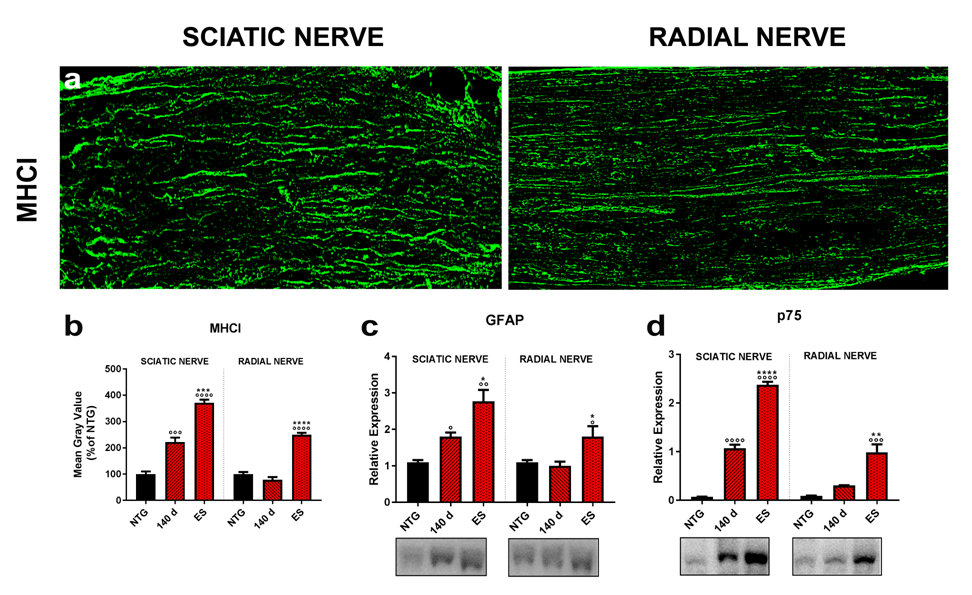
**

**Supplementary Figure 12. Regional and temporal differences defines the disease progression of mSOD1 mice.** (**a**) Confocal micrographs of longitudinal sections of the sciatic nerve and radial nerves of G93A+/+ mice at the endstage (ES) showing the level of expression of MHCI in the sciatic nerves and in radial nerves. Bar, 100 μm. (**b**) Quantification of MHCI immunoreactivity (mean gray value) in sciatic and radial nerves of G93A+/+ mice compared to NTG littermates at 140 d and ES. The analysis was done on radial and sciatic nerves of the same animals. Data are expressed as the percentage of relative NTG (mean ± SEM) from three independent experiments (at least four serial sections for each animal) for each genotype. ^°°°^P < 0.001; ^°°°°^P < 0.0001 (140 d; ES vs NTG); ^***^P < 0.001; ^****^P < 0.0001 (ES vs 140 d) by one-way ANOVA with Tukey’s post analysis. (**c, d**) Densitometric analysis of immunoblots of (c) GFAP and (d) p75^NTR^ in sciatic and radial nerve extracts from NTG and G93A+/+ mice at 140 d and ES. Immunoreactivity was normalized the total amount of protein detected by red Ponceau (not showed). The analysis revealed an earlier activation of both GFAP and p75^NTR^ in the sciatic nerves than in the radial nerve of G93A+/+ mice. Data are reported as the percentage of relative NTG (mean ± SEM) from three independent experiments for NTG and G93A+/+ mice. ^°^P < 0.05; ^°°^P < 0.01; ^°°°^P < 0.001; ^°°°°^P < 0.0001 (140 d; ES vs NTG); ^*^P < 0.05; ^**^P < 0.01; ^****^P < 0.0001 (ES vs 140 d) by one-way ANOVA with Tukey’s post analysis.
